# Depending on the stress, histone deacetylase inhibitors act as heat shock protein co-inducers in motor neurons and potentiate arimoclomol, exerting neuroprotection through multiple mechanisms in ALS models

**DOI:** 10.1007/s12192-019-01064-1

**Published:** 2020-01-03

**Authors:** Rachel Kuta, Nancy Larochelle, Mario Fernandez, Arun Pal, Sandra Minotti, Michael Tibshirani, Kyle St. Louis, Benoit J. Gentil, Josephine N. Nalbantoglu, Andreas Hermann, Heather D. Durham

**Affiliations:** 1 Department of Neurology and Neurosurgery and Montreal Neurological Institute, McGill University, 3801 University St., Montreal, QC H3A 2B4 Canada; 2 Department Neurology, Technische Universität Dresden, Fetscherstr. 74, 01307 Dresden, Germany; 3 Translational Neurodegeneration Section “Albrecht-Kossel”, Department of Neurology and Center for Transdisciplinary Neuroscience (CTNR), University Medical Center Rostock, University of Rostock, Rostock, Germany and German Center for Neurodegenerative Diseases (DZNE) Rostock, Rostock, Germany

**Keywords:** Heat shock response, Motor neuron, Hsp70, HSPA1A, Histone deacetylase inhibitor, DNA repair, SOD1, FUS, HSP90 inhibitor, Amyotrophic lateral sclerosis, Motor neuron disease, Arimoclomol

## Abstract

Upregulation of heat shock proteins (HSPs) is an approach to treatment of neurodegenerative disorders with impaired proteostasis. Many neurons, including motor neurons affected in amyotrophic lateral sclerosis (ALS), are relatively resistant to stress-induced upregulation of HSPs. This study demonstrated that histone deacetylase (HDAC) inhibitors enable the heat shock response in cultured spinal motor neurons, in a stress-dependent manner, and can improve the efficacy of HSP-inducing drugs in murine spinal cord cultures subjected to thermal or proteotoxic stress. The effect of particular HDAC inhibitors differed with the stress paradigm. The HDAC6 (class IIb) inhibitor, tubastatin A, acted as a co-inducer of Hsp70 (HSPA1A) expression with heat shock, but not with proteotoxic stress induced by expression of mutant SOD1 linked to familial ALS. Certain HDAC class I inhibitors (the pan inhibitor, SAHA, or the HDAC1/3 inhibitor, RGFP109) were HSP co-inducers comparable to the hydroxyamine arimoclomol in response to proteotoxic stress, but not thermal stress. Regardless, stress-induced Hsp70 expression could be enhanced by combining an HDAC inhibitor with either arimoclomol or with an HSP90 inhibitor that constitutively induced HSPs. HDAC inhibition failed to induce Hsp70 in motor neurons expressing ALS-linked mutant FUS, in which the heat shock response was suppressed; yet SAHA, RGFP109, and arimoclomol did reduce loss of nuclear FUS, a disease hallmark, and HDAC inhibition rescued the DNA repair response in iPSC-derived motor neurons carrying the FUS^P525L^mutation, pointing to multiple mechanisms of neuroprotection by both HDAC inhibiting drugs and arimoclomol.

**Electronic supplementary material:**

The online version of this article (10.1007/s12192-019-01064-1) contains supplementary material, which is available to authorized users.

## Introduction

Protein misfolding and aggregation are associated with multiple neurodegenerative disorders including amyotrophic lateral sclerosis (ALS). Mutations, post-translational modifications or increased protein concentration, coupled with the biophysical properties of the affected protein(s), increase demands on chaperoning and proteolytic mechanisms. A logical therapeutic strategy is to boost the chaperoning capacity of neural cells by inducing heat shock proteins (HSPs) to manage the load of aberrant proteins and maintain function; however, certain challenges must be overcome. Many types of neurons, including motor neurons, have a high threshold for increasing expression of HSPs in response to stress (Batulan et al. 2003; Manzerra and Brown 1992) and are relatively resistant to co-inducers of HSP expression, drugs that magnify an existing stress response (Batulan et al. 2005). In addition, disease processes can inhibit the response to HSP-inducers, with loss of efficacy occurring subsequent to changes in chromatin architecture (Labbadia et al. 2011).

Eukaryotic expression of HSPs is mediated by binding and activation of heat shock transcription factors (HSFs) to heat shock elements (HSEs) on *HSP* promoters. Environmental and physiological stresses activate transcription of *HSP* genes largely through HSF1 (Morimoto 1998). Monomeric HSF1 is sequestered in a multichaperone complex including HSP90, HSP70, P97/VCP, HDAC6, and cofactors. Upon stress, misfolded proteins compete for chaperones and HSF1 is released, trimerizes and binds to HSEs. HSF1 is subject to multiple post-translational modifications, including phosphorylation, sumoylation, ubiquitination, and acetylation, which regulate DNA binding, transactivation of heat shock genes and degradation (Boyault et al. 2007; Dayalan Naidu and Dinkova-Kostova 2017; Joutsen and Sistonen 2019; Li et al. 2017; Pernet et al. 2014). Whereas phosphorylation of residues in HSF1’s regulatory domain was thought to be required for transactivational competence, more recent evidence points to a role in fine tuning of the heat shock response, including regulation of HSF1 binding to promoter elements (Budzynski et al. 2015). Another regulatory factor is the translation elongation factor eEF1A1, which mediates stress-induced *HSP70* (*HSPA1A*) transcription as well as stability and transport of *HSP70* mRNA (Vera et al. 2014). Motor neurons exhibit an underlying reticence for stress-induced activation of HSF1 (Batulan et al. 2003) and the neuron-specific variant eEF1A2 lacks the regulatory ability of eEF1A1 (Vera et al. 2014).

Acetylation has multiple and sometimes opposite effects on aspects of the heat shock response, including HSF1 regulation. Acetylation by EP300/CREBBP stabilizes HSF1 under homeostatic conditions, whereas additional acetylation during thermal stress dampens the heat shock response by releasing HSF1 from HSE, an effect that is antagonized by deacetylation by SIRT1 (Raychaudhuri et al. 2014; Westerheide et al. 2009). Acetylation of HSP90 by HDAC6 suppresses its chaperone function (Bali et al. 2005).

Histone acetylation and the chromatin landscape influence expression of heat shock genes. The fundamental structure of chromatin is the nucleosome, composed of an octameric complex of the core histone proteins, H1, H2A, H2B, H3, and H4. In general, acetylation of histones is permissive to gene expression by opening up chromatin to permit access of transcription factors to gene promoters, whereas deacetylation is suppressive. The level of acetylation is regulated by histone acetyl transferases and histone deacetylases. With respect to the stress-inducible binding of HSF1 to HSE of heat shock genes, binding occurs at areas of open chromatin with tetra-acetylated H4 and acetylated H3K9 marks (Guertin and Lis 2010). In the R6/2 mouse model of Huntington’s disease, attenuation of the efficacy of the HSP-inducing drug, HSP990, was linked to reduced levels of tetra-acetylated histone H4 (Labbadia et al. 2011). The chaperone co-inducer BGP-15 increased chromatin accessibility at multiple loci, including *HSPA1A*, and lowered the threshold for activation of Hsf1 in heat shocked mouse embryonic fibroblasts. This effect was associated with reduced histone deacetylase (HDAC) activity in the cells, which would increase histone acetylation (Budzynski et al. 2017). However, it is not clear how these epigenetic mechanisms contribute to regulation of heat shock genes and stress response in neurons.

Histone acetylation regulates HSE accessibility by recruiting chromatin remodeling complexes (CRC). In yeast, these CRC are referred to as SWI/SNF complexes. Hsf1 recruits Brg1, the ATPase/helicase component of SWI/SNF, to remodel chromatin for transcription and elongation at heat shock genes (Calderwood et al. 2010; Shivaswamy and Iyer 2008; Sullivan et al. 2001). Brg1 interacts with nucleosomes through binding of its bromodomain to acetylated lysines of histone tails, acetylated lysine 14 of histone 3 (H3K14ac) being the favored substrate (Shen et al. 2007). In vertebrates, SWI/SNF is known as Brahma-related gene 1 (Brg1)-associated factor complex (BAF) (Vogel-Ciernia and Wood 2013). A regulated switch of key subunits to neuronal isoforms to form neuronal BAF complexes (nBAF) is a key requirement for expressing gene sets for neuronal differentiation, including process extension (Wu et al. 2007). We identified loss of nBAF CRC proteins as a pathway common to multiple types of ALS including both familial and sporadic disease (Tibshirani et al. 2017). Nuclear Brg1/SMARCA4 and other key nBAF components (Baf53b and CREST) were depleted from cultured murine motor neurons expressing FUS or TDP-43 mutants linked to familial ALS (fALS) and in spinal motor neurons of fALS (SOD1^A4V^, C9ORF72) and sporadic ALS (sALS) autopsy specimens (Tibshirani et al. 2017). There is also evidence of reduced histone acetylation in ALS (Cudkowicz et al. 2009; Janssen et al. 2010; Liu et al. 2013) and in transgenic mouse models of fALS1 due to *SOD1* mutations (Rouaux et al. 2003; Ryu et al. 2005).

Thus, various epigenetic changes could impair the ability of neurons to protect themselves by upregulating neuroprotective stress pathways, including HSPs to chaperone misfolded proteins for degradation and attenuate the heat shock response in chronic neurodegenerative disease. In this study, we determined whether inhibitors of different HDAC classes would enable the heat shock response in motor neurons and would improve the efficacy of HSP-inducing drugs in experimental models relevant to ALS, using four experimental paradigms:

1. Induction of Hsp70 by the HSP90 inhibitor, NXD30001: HSP90 inhibitors constitutively induce expression of HSPs by disrupting HSP90 complexes; HSP90 also participates in the removal of HSF1 trimers from HSE, such that HSP90 inhibitors prolong the HSF1-HSE interaction (Kijima et al. 2018). Although of interest in therapy of neurodegenerative disorders, HSP90 inhibitors have issues of toxicity and CNS bioavailability, having been largely developed as cancer chemotherapeutic agents. Nevertheless, these drugs are highly useful for proof-of-principle studies, particularly in culture. The HSP90 inhibitor, NXD30001, is highly effective in spinal cord cultures, upregulating Hsp70 expression, particularly in neurons (Cha et al. 2014). The serendipitous discovery that the pan HDAC inhibitor, SAHA, quite strikingly increased Hsp70 expression initiated by NXD30001 led us to envisage the present study.

2. The limited response of neurons to express HSP70 after heat shock: We previously reported that motor neurons in spinal cord-DRG cultures have a high threshold for induction of a stress response in response to heat shock, such that thermal stress induces little or no Hsp70 (Batulan et al. 2003), thus providing a good model to investigate how HDAC inhibitors might overcome the high threshold for induction.

3. Proteotoxic stress induced by the expression of mutant SOD1 linked to familial ALS1: As a proteotoxic stress relevant to neurodegenerative disease, we used our previously established model of ALS1 (Durham et al. 1997) due to mutations in *SOD1*, in which upregulation of HSP expression alleviated many aspects of mutant SOD1 toxicity in cultured motor neurons (Batulan et al. 2006; Cha et al. 2014).

4. Proteotoxic stress induced by expression of mutant FUS linked to familial ALS6, in which histone acetylation is compromised: A number of genes linked to ALS encode RNA binding proteins, including FUS (Kwiatkowski Jr. et al. 2009; Vance et al. 2009). In our primary culture model (Tradewell et al. 2012), histone acetylation is impaired in motor neurons expressing mutant FUS, but preserved by treatment with the pan HDAC inhibitor, SAHA (Tibshirani et al. 2015), thus providing a model to examine the heat shock response in light of relevant epigenetic abnormalities.

Use of these models revealed that different classes of HDAC inhibitors act as HSP co-inducers depending on the type of stress and enhance co-induction of Hsp70 by arimoclomol in multiple scenarios. In addition, they preserve DNA repair and promote nuclear retention of mutant FUS through non-HSP mechanisms.

## Materials and methods

### Spinal cord-dorsal root ganglion (DRG) cultures

Spinal cord-DRG cultures were prepared and cultured as previously described (Durham et al. 1997; Roy et al. 1998). Briefly, spinal cords with attached DRG were dissected from E13 CD1mouse embryos, dissociated using trypsin, and plated on poly-lysine and Matrigel-coated coverslips. Cultures were maintained in modified N3 hormone and growth factor-supplemented culture medium with 1–2% horse serum and used for experiments 3–8 weeks after plating. At this time, motor neurons were identified morphologically by their large size and extensive dendritic tree, as previously documented (Roy et al. 1998).

### Heat shock

For heat shock, spinal cord-DRG cultures on coverslips were transferred to 35 mm culture dishes containing 2 ml of buffer (minimum essential medium enriched with 5 g glucose without sodium bicarbonate; pH 7.4). The dishes were wrapped in Parafilm and placed in a water bath at 43 °C for 30 min, with temperature monitoring, following which cultures were returned to culture medium including drug treatments and recovered for 6 to 72 h in the incubator at 37 °C in 5% CO_2_.

### Expression of ALS-linked mutant genes

Culture models of ALS1, due to mutation in *SOD1*, and ALS6, due to mutation in *FUS*, were prepared as previously described (Durham et al. 1997; Tradewell et al. 2012) by microinjection of expression plasmids into motor neuronal nuclei. Plasmids used in this study were N-terminal Flag-tagged *FUS*^*R521H*^ in pcDNA3 microinjected at 20 ng/μl (Tradewell et al. 2012) and *SOD1*^*G93A*^ in pCEP4 microinjected at 200 ng/μl (Durham et al. 1997). Microinjected neurons were identified by including 2.5 μg/μl 70 kDa dextran-FITC in the injectate (Molecular Probes Inc., Eugene, OR) or by expressing mCherry from mCherry-C1 plasmid (Clontech #632524), coinjected at 1–2 ng/μl.

### Immunocytochemistry

Cultures were fixed in 4% paraformaldehyde (PFA) in PBS for 10 min, permeabilized in 0.5% NP-40 in PBS for 1 min, and fixed again in PFA for 2 min. Cultures were placed in blocking solution (5% horse serum in PBS) for either 30 min (at room temperature) or overnight (at 4 °C) to prevent non-specific binding of antibodies. Cultures were incubated with primary or secondary antibodies diluted in blocking solution for 30 min, with three 5 min washes in PBS after each antibody. For histochemical detection, cultures were treated with 0.6% H_2_O_2_ in PBS for 2 min following primary antibody incubation. Horse radish peroxidase (HRP)-conjugated secondary antibody was detected by 5-min exposure to 0.03% ImmPACT DAB Chromogen dissolved in ImmPACT DAB diluent (Vector Laboratories, USA) followed by three 5 min washes in PBS. Coverslips were mounted onto microscope slides using Immumount (ThermoFisher Scientific, Canada). Cells were visualized using a Zeiss Observer Z1 microscope (Carl Zeiss Canada Ltd.) equipped with epifluorescence optics and a Hamamatsu ORCA-ER cooled CD camera (Hamamatsu, Japan). Images were acquired with Zeiss Axiovision software.

To score expression of Hsp70 in motor neurons exposed to the various treatments, a semiquantitative method was used. The primary outcome was absence (0) or presence of immunolabeling (+), given that very few motor neurons express this protein under normal conditions or in response to some stressors. In certain experiments, the relative intensity of Hsp70 was scored as + or ++. To establish the reproducibility of the scoring method, two individuals independently scored the experiment; there was no statistical difference between their scores.

### Western analysis

Cultures were collected in protein lysis buffer (50 mM Tris-HCl pH 6.8, 2% SDS 10% glycerol and supplemented with protease inhibitor cocktail (Sigma-Aldrich Canada, #P8340) and samples were separated by 12–15% SDS-PAGE. After transfer to nitrocellulose, membranes were blocked in 5% skim milk in TRIS-buffered saline (TBS) plus Tween 20 0.05% (TBST) and probed with primary antibodies and HRP-conjugated secondary antibodies in 5% skim milk, interspersed with three 10 min washes in TBST. Labeling was detected using HyGlo Chemiluminescent HRP antibody detection reagent (Denville Scientific, USA). Images were acquired with an Intas imaging System (Intas GmbH, Germany), and densitometry of bands was performed using NIH ImageJ software. Levels of acetylated H3K9/K14 and Hsp70 were normalized to GAPDH and acetylated tubulin was normalized to α-tubulin, followed by normalization to the vehicle-treated control.

### Antibodies and chemicals

Primary antibodies were: mouse anti-human HSP70, specific for stress-inducible HSPA1A (StressMarq, Canada, SMC-100B; 1:100 ICC; 1:1000 WB), mouse anti-Flag M2 (Sigma-Aldrich, #F1804; 1:400 ICC); rabbit anti-FUS (Proteintech, USA, 11570-1-AP; 1:400 ICC), mouse anti-human SOD1 (Sigma-Aldrich Canada, SD-G6; 1:100 ICC), rabbit anti-acetyl-histone H3K9/K14 (Cell Signaling, #9677; 1:400 ICC; 1:1000 WB), mouse anti-GAPDH (MediMabs, Canada, #MM-0163; 1:1000 WB), moue monoclonal anti-acetylated tubulin (Sigma-Aldrich #T6793; 1:1000 WB) and rabbit anti-α-tubulin (Abcam #ab15246; 1:1000 WB).

Secondary antibodies (Jackson Immunoresearch: Cedarlane, Canada): Alexa Fluor 488-conjugated Affinipure donkey anti-mouse IgG (1:300); Cy3-conjugated donkey anti-mouse IgG 1:300); Cy5-conjugated donkey anti-rabbit IgG (1:300), and HRP-conjugated goat anti-mouse (1/5000 for WB, 1/500 for IC) or donkey anti-rabbit IgG (Jackson Immunoresearch (1:2500).

HDAC inhibitors and HSP-inducing drugs: SAHA (suberoylanilide hydroxamic acid) and tubastatin A (Cayman Chemical: Cedarlane, Canada); tacedinaline, RGFP109 and RGFP966 (Selleckchem: Cedarlane, Canada); sodium phenylbutyrate (Selleckchem); arimoclomol (Toronto Research Chemicals, Canada); NXD30001 was previously supplied by NexGenix Pharmaceuticals (Cha et al. 2014).

### DNA damage assay in human NPC-derived motor neurons

The generation of human neuronal progenitor cells (NPCs) and motor neurons was accomplished by following the protocol of Reinhardt et al. (Reinhardt et al. 2013). All lines were established previously (Naumann et al. 2018; Pal et al. 2018) and in accordance with the local ethics committee of the Technische Universität Dresden, Germany (EK45022009).iPSC-derived spinal motor neurons were matured in 3.5-cm glass bottom dishes coated with poly-L-ornithine and laminin (Reinhardt et al. 2013) over 21 days in vitro (DIV) and then treated for 24 h with Mock (0.1% DMSO), 4 μM RGFP109, 0.1 μM tubastatin A or 2 μM SAHA, all from a stock in DMSO.

Recruitment of FUS-GFP to laser irradiation sites (linear cuts) was then performed using a custom-build 355 nm UV laser cutter setup similar to common commercial FRAP modules with a sharply focused beam in conjunction with a motorized stage and a piezo-electric Z-drive for precise 3D-targeting. For detailed technical specifications, refer to (Naumann et al. 2018). A Zeiss alpha Plan-Fluar 100 × 1.45 oil immersion objective was used and 24 laser shots in 0.5 μm-steps were administered to generate a 12 μm linear cut through nuclei. Recruitment and release of FUS-GFP at laser irradiation sites (on-off kinetics in boxed areas in Fig. 9a) was recorded with 1 fps over 15 min in confocal live spinning disk mode using the 488 nm laser line for excitation of GFP. In order to deduce on-off kinetics, a narrow rectangle selection box was drawn in FIJI software around the cut in the acquired movies and integrated intensities obtained with the FIJI plugin “Analyze/Plot Profile” were normalized by the nuclear background using a selection box of identical dimensions. The resultant fold-change values were plotted over consecutive movie frames (i.e., time) to generate Fig. 9b.

### Statistical analysis

The analyses for each experiment are described in the figure legends. Significance was established at *p* ≤ 0.05.

## Results

### HDAC inhibitors: dose-response analysis in spinal cord-DRG cultures

Major mammalian HDACs are classified as class I (HDAC 1, 2, 3, 8), class IIa (HDAC4, 5, 7, 9) and IIb (HDAC6, 10), class III (the sirtuins), and class IV (HDAC11). Of particular interest to this study were class I and IIb: class I HDACs because of their importance for histone acetylation and chromatin remodeling; class IIb HDAC6 for specific effects on tubulin acetylation, microtubule stability and intracellular transport, as well as its regulatory role on the duration of HSF1 activation and chaperone function of HSP90 (Bali et al. 2005; Boyault et al. 2007; Pernet et al. 2014).

**Fig. 1:**
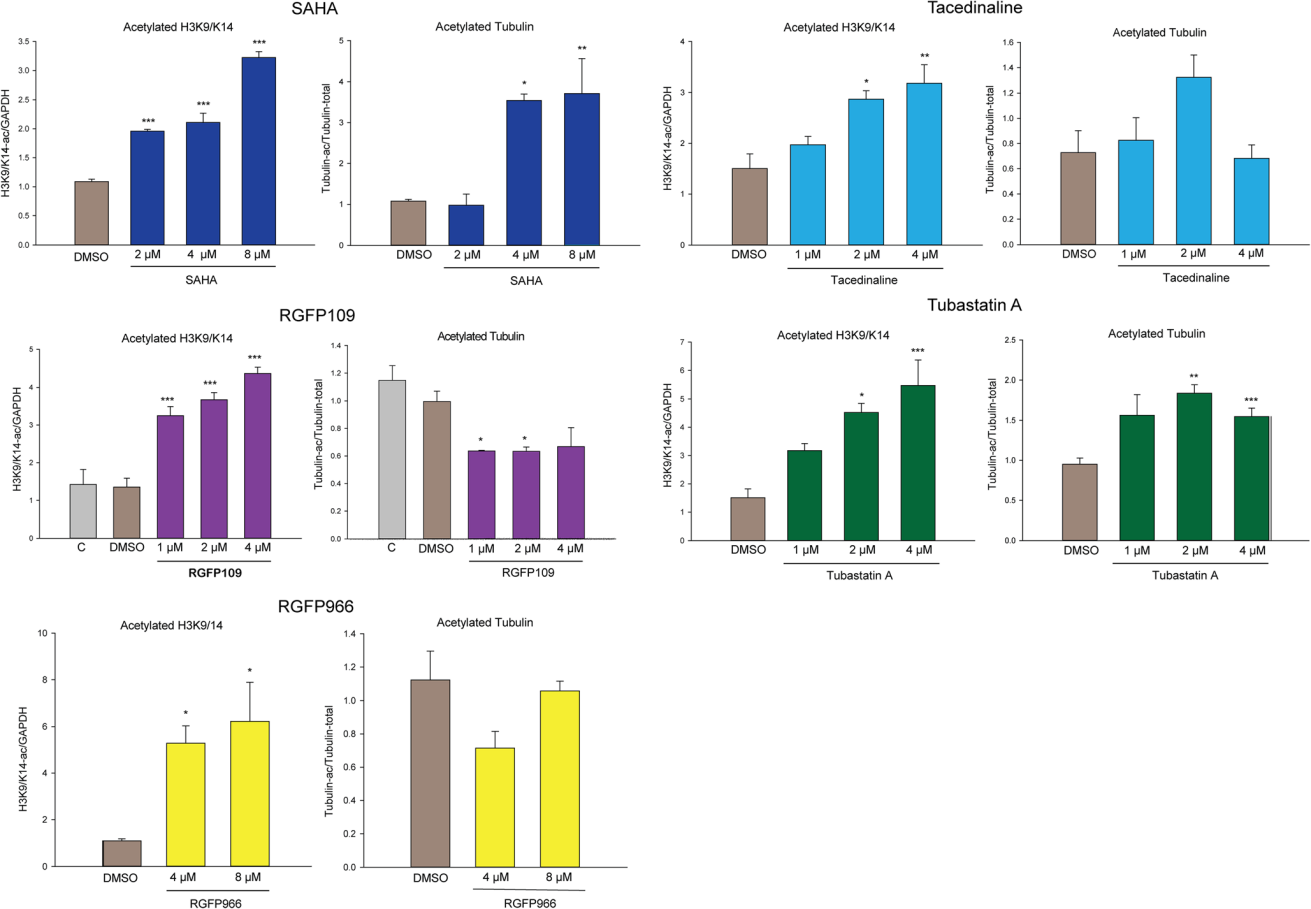
Dose-response of various HDAC inhibitors on histone and α-tubulin acetylation in murine dissociated spinal cord-DRG cultures, as summarized in Table 1. Four- to 6-week-old cultures were treated for 24 h with each of the following HDAC inhibitors: SAHA (pan HDAC inhibitor), tacedinaline (HDAC 1 inhibitor), RGFP966 (HDAC 3 inhibitor), RGFP109 (HDAC1/3 inhibitor), and tubastatin A (HDAC6 inhibitor). Protein samples were separated by 15% SDS-PAGE and transferred to nitrocellulose membranes. Western analysis was carried out using antibodies to acetyl-histone H3K9/K14, GAPDH, acetylated tubulin, and total α-tubulin as described in “Materials and methods.” Density of bands corresponding to acetylated H3 and acetylated tubulin were normalized to GAPDH and α-tubulin, respectively, and values from treated samples were then normalized to vehicle (DMSO)-treated controls. Plotted are means ± SEM of data from 3 cultures per condition. Statistical significance was evaluated by one-way ANOVA and Dunnett post hoc analysis: *p < 0.05, **p < 0.01, ***p < 0.001, ****p < 0.0001

**Fig. 2:**
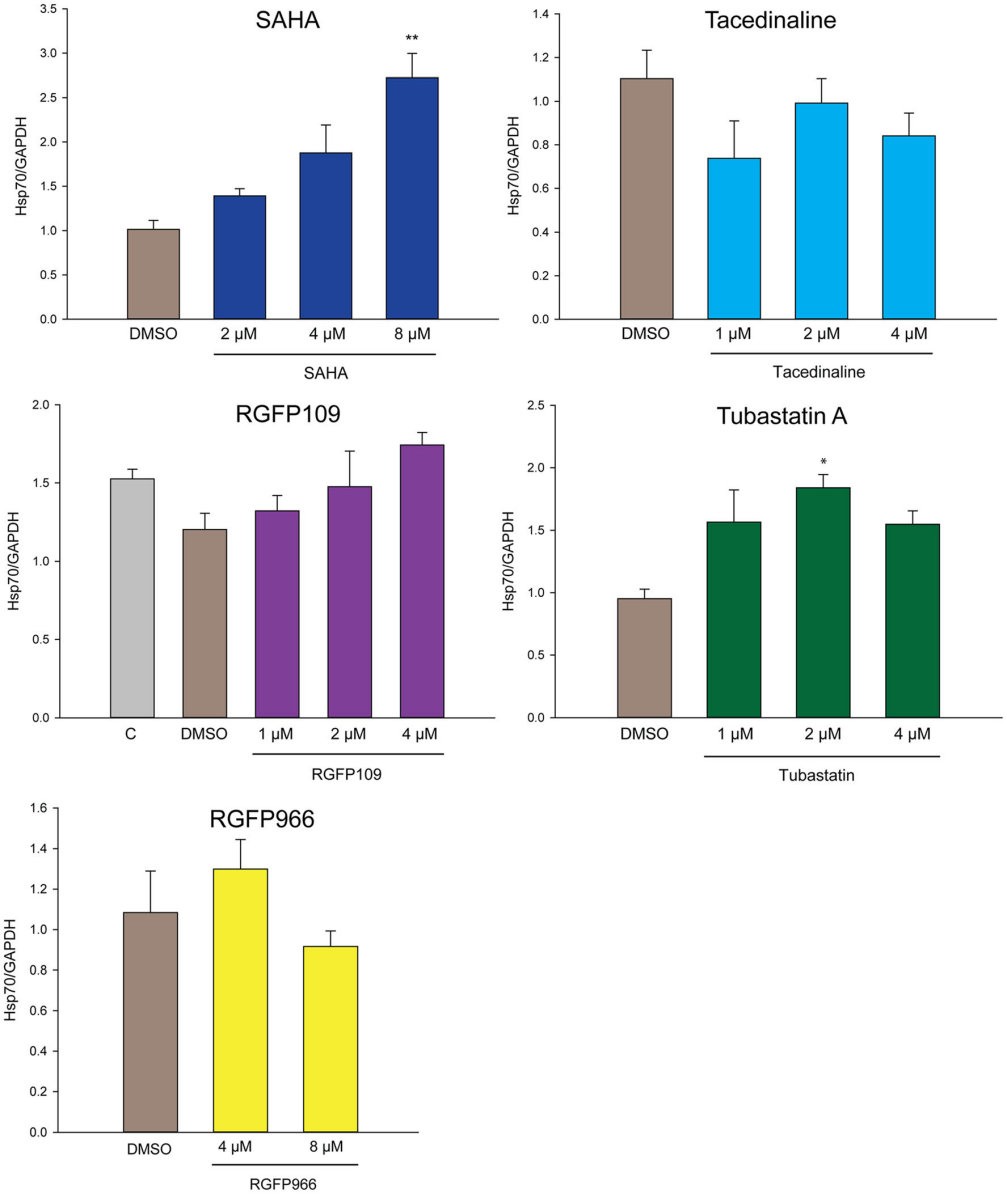
Dose-response of various HDAC inhibitors on expression of stress-inducible Hsp70 (HSPA1A) in dissociated spinal cord-DRG cultures. Cultures were treated for 24 h with each of the following HDAC inhibitors: SAHA (pan HDAC inhibitor), tacedinaline (HDAC 1 inhibitor), RGFP966 (HDAC 3 inhibitor), RGFP109 (HDAC1/3 inhibitor), and tubastatin A (HDAC6 inhibitor). Protein samples were separated by 12% SDS-PAGE and transferred to nitrocellulose membranes. Western analysis was carried out using antibodies to Hsp70 and GAPDH, as described in “Materials and methods.” Densities of bands corresponding to Hsp70 were normalized to GAPDH, and values from treated samples were then normalized to vehicle (DMSO)-treated controls. Plotted are means ± SEM of data from 3 cultures per condition. Statistical significance was evaluated by one-way ANOVA with Dunnett post-hoc analysis: *p < 0.05, **p < 0.01, ***p < 0.001, ****p < 0.0001

Suberanilohydroxamic acid (SAHA, Vorinostat®), and phenylbutyrate have relatively broad substrate specificity. We tested SAHA as a standard pan HDAC inhibitor and conducted limited experiments with phenylbutyrate. To associate effects with particular HDAC types, experiments were conducted with inhibitors of HDAC1 (tacedinaline), HDAC3 (RGFP966), HDAC1 and 3 (RGFP109) and HDAC6 (tubastatin A). Figure 1 shows the Western blot analysis of dose-response of spinal cord cultures treated with drug for 24 h, measuring acetylation of histone 3 (acetylated at residues K9 and K14 (H3K9/K14)) and acetylated α-tubulin as experimental endpoints, referenced to GAPDH or total α-tubulin, respectively. Figure 2 shows the dose–response for expression of stress-inducible Hsp70 (HSPA1A). Western blots for Fig. 1 and Fig. 2 are presented in Online Resource 1 and data are summarized in Table 1. Drug concentrations were chosen for further study that significantly increased acetylation of target substrate. The following data are summarized in Table 2.

**Table 1:** Summary of HDAC inhibitors analyzed and effect on histone and tubulin acetylation presented in Fig. 1. SAHA, RGFP109 and tubastatin A were selected for detailed study, as explained in the text

HDAC inhibitor concentration	HDAC	Increased H3K9/K14Ac and H4Ac	Increased tubulin acetylation
SAHA 2–4 μm	Pan	Yes	At 4 μM
Phenylbutyrate 1–3 mM	Pan	Yes	–
RGFP109 4 μM	1 and 3	Yes	No
RGFP966 4 μM	3	Yes	No
Tacedinaline 4 μM	1	Yes	No
Tubastatin A 1–2 μM	6	Slight at 2 μM	Yes

### HDAC inhibition potentiates NXD30001-induced expression of Hsp70

**Fig. 3:**
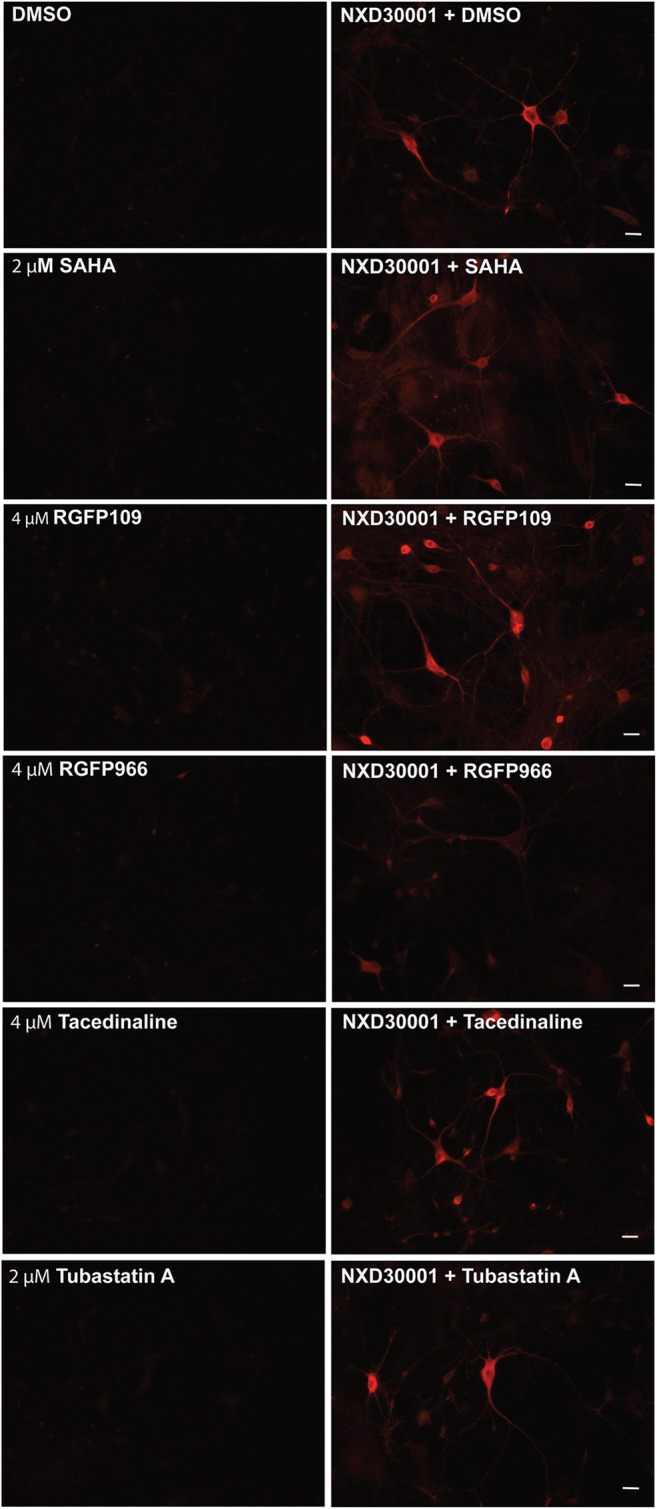
HDAC inhibitors enhanced expression of Hsp70 induced by the Hsp90 inhibitor, NXD30001. Cultures were treated for 3 days with 40 nM NXD30001 or vehicle (DMSO), alone or in combination with an HDAC inhibitor—2 μM SAHA (pan HDAC inhibitor), 4 μM tacedinaline (HDAC 1 inhibitor), 4 μM RGFP966 (HDAC 3 inhibitor), 4 μM RGFP109 (HDAC1/3 inhibitor), or 2 μM tubastatin A (HDAC6 inhibitor). Cultures were fixed and immunolabeled with antibody specific for stress-inducible Hsp70 (HSPA1A). All HDAC inhibitors except RGFP966 enhanced NXD30001-induced Hsp70 expression, but had no inducing effect on their own (See Fig. 4 for quantitation). SAHA and RGFP109 also enhanced NXD30001-induced Hsp70 expression in background glial cells. Scale bar = 20 μm

**Fig. 4:**
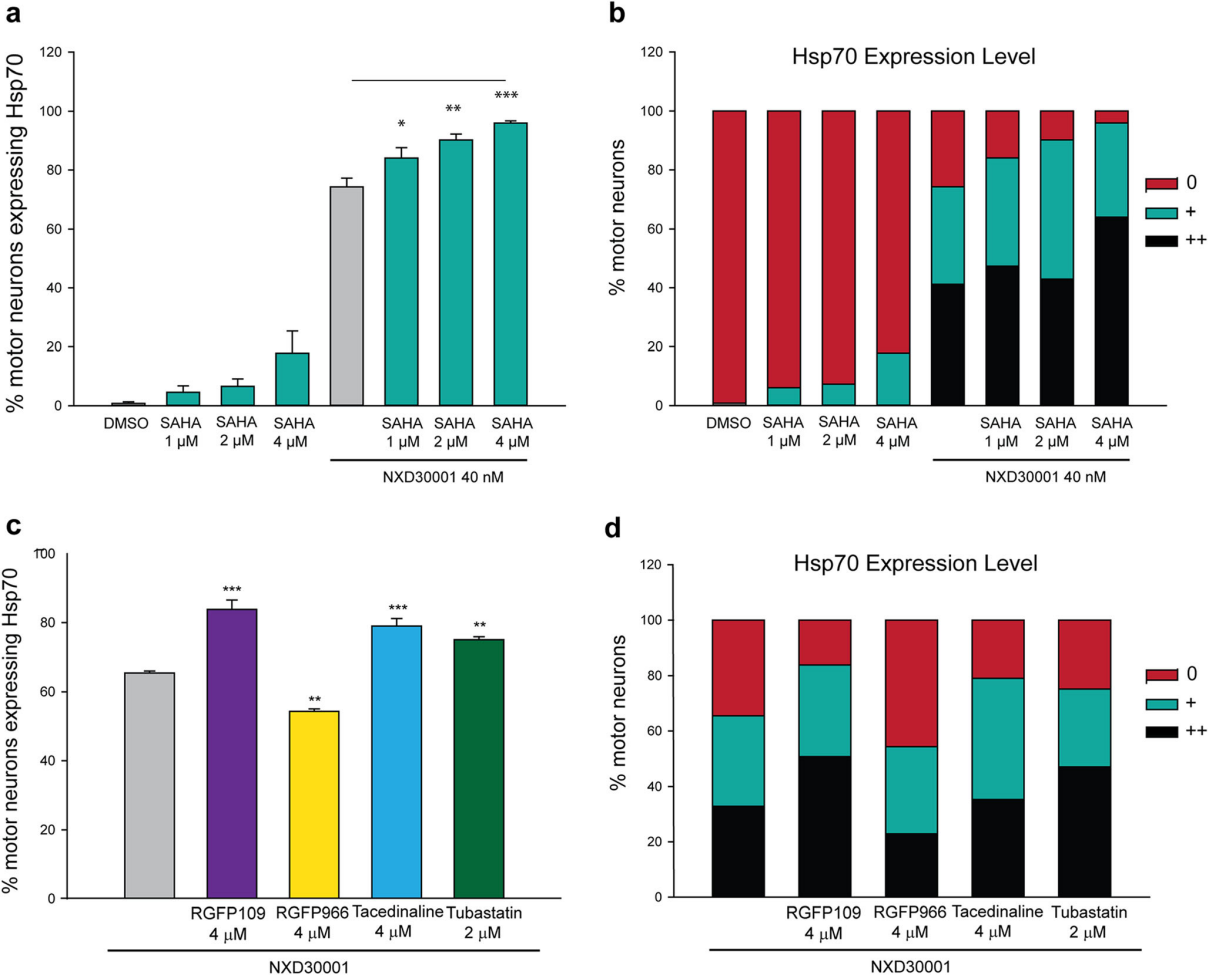
HDAC inhibitors enhanced expression of Hsp70 induced by the Hsp90 inhibitor, NXD30001. Cultures were treated for 3 days with 40 nM NXD30001 or vehicle (DMSO), alone or in combination with an HDAC inhibitor: **a**, **b** 1–4 μM SAHA (pan HDAC inhibitor), or **c**, **d** 4 μM tacedinaline (HDAC 1 inhibitor), 4 μM RGFP966 (HDAC 3 inhibitor), 4 μM RGFP109 (HDAC1/3 inhibitor) or 2 μM tubastatin A (HDAC6 inhibitor). Cultures were fixed and immunolabeled with antibody specific for stress-inducible Hsp70 (HSPA1A) (see representative micrographs shown in Fig. 3). Hsp70 expression was scored in at least 75 motor neurons per culture as 0 (background), + (light) or ++ (strong) and graphed as mean ± SEM of at least 3 cultures per condition. All HDAC inhibitors except RGFP966 enhanced NXD30001-induced Hsp70 expression, increasing the percentage of motor neurons expressing and the intensity of immunolabeling, but had no inducing effect on their own (as shown for SAHA). Statistical analysis was performed using one-way ANOVA followed by Dunnett’s multiple comparison test to NXD control: *p < 0.05, **p < 0.01, ***p < 0.001

HSP90 inhibitors constitutively induce expression of HSPs in the absence of additional stress. The small molecule NXD30001, previously shown to induce HSP expression in cultured motor neurons (Cha et al. 2014), was used to determine which HDAC inhibitors would potentiate constitutive HSP-inducers. Hsp70 expression was examined by immunocytochemistry after 3 days of treatment with 40 nM NXD30001 or vehicle control. Representative epifluorescence images are presented in Fig. 3. Hsp70 labeling was scored as negative (0 – relative to second antibody control), light (+) or bright (++) and quantified as the percentage of total motor neurons counted (minimum of 75 neurons scored in each of at least 3 cultures) (Fig. 4). The pan HDAC inhibitor, SAHA, increased both the percentage of NXD30001-treated motor neurons expressing Hsp70 (Fig. 4a) and the intensity of labeling (Fig. 4b) in a dose-related manner, at concentrations of 1–4 μM, which alone did not significantly alter Hsp70 expression. RGFP109 (HDAC1/3 inhibitor), tacedinaline (HDAC1 inhibitor), and tubastatin A (HDAC6 inhibitor) also potentiated NXD30001-induced Hsp70, whereas the HDAC 3 inhibitor, RGFP966 was suppressive (Fig. 4c, d). Activity of RGFP109 was the most similar to SAHA in this assay, as both inhibitors potentiated Hsp70 expression in background glial cells as well as in neurons (see images in Fig. 3).

**Table 2:** Summary of the effect of HDAC inhibitors on the experimental endpoints assayed

HDA inhibitor concentration	HDAC	Enhanced HSP induction by NXD30001		Enhanced HSP induction by heat shock	Enhanced HSP induction by mutant SOD1	Enhanced HSP induction by mutant FUS	Preserved nuclear mutant FUS in motor neurons	DNA Repair in mutant FUS motor neurons
		Motor neurons	Non neuronal					
SAHA 2–4 μM	Pan	Yes	Yes	Yes	Yes	No	Yes	Yes
Phenylbutyrate 1–3 mM	Pan	No	No	No	–	–	–	–
RGFP109 4 μM	1 and 3	Yes	Yes	Yes	Yes	No	Yes	Yes
RGFP966 4 μM	3	No	No	–	–	–	Yes	–
Tacedinaline 4 μM	1	Yes	Yes	–	–	–	No	–
Tubastatin A 1–2 μM	6	Yes	Light	Yes	No	No	No	Yes

### HDAC inhibition potentiates arimoclomol-mediated enhancement of Hsp70 expression by heat shock

Based on the collective experimental data, SAHA, RGFP109, and tubastatin A were selected for further study. Tubastatin A was of interest because HDAC6 inhibitors have shown beneficial effects in models of Charcot-Marie-Tooth peripheral neuropathies (Benoy et al. 2018; d'Ydewalle et al. 2011) and restored axonal transport in motor neurons derived from induced pluripotent stem cells (iPSC) from ALS patients with *FUS* mutation (Guo et al. 2017). Although tacedinaline showed some efficacy in the NXD30001 study, it was not protective in a model of ALS due to mutation in *FUS*, presented in Fig. 8 and therefore was not pursued further.

Arimoclomol acts by promoting HSF1 binding to HSE elements of heat shock genes (Kalmar and Greensmith 2009; Kieran et al. 2004), but does not induce HSPs in the absence of stress, including in our spinal cord-DRG cultures (not shown). To determine if HDAC inhibitors potentiate arimoclomol, we used nonlethal heat shock as the initiating stress (43 °C for 30 min, as previously described (Batulan et al. 2003)). Arimoclomol’s dose-response was investigated in preliminary experiments (Online Resource 2), in which cultures were exposed to arimoclomol (1–10 μM) or vehicle beginning 30 min prior to heat shock and the percentage of motor neurons expressing Hsp70 was quantified after recovery in the original drug-containing medium for 6, 24, 48, or 72 h. Arimoclomol significantly enhanced Hsp70 expression (relative to heat shocked vehicle control) at 6- and 24-h recovery times; the effect was absent by 48-h recovery.

**Fig. 5:**
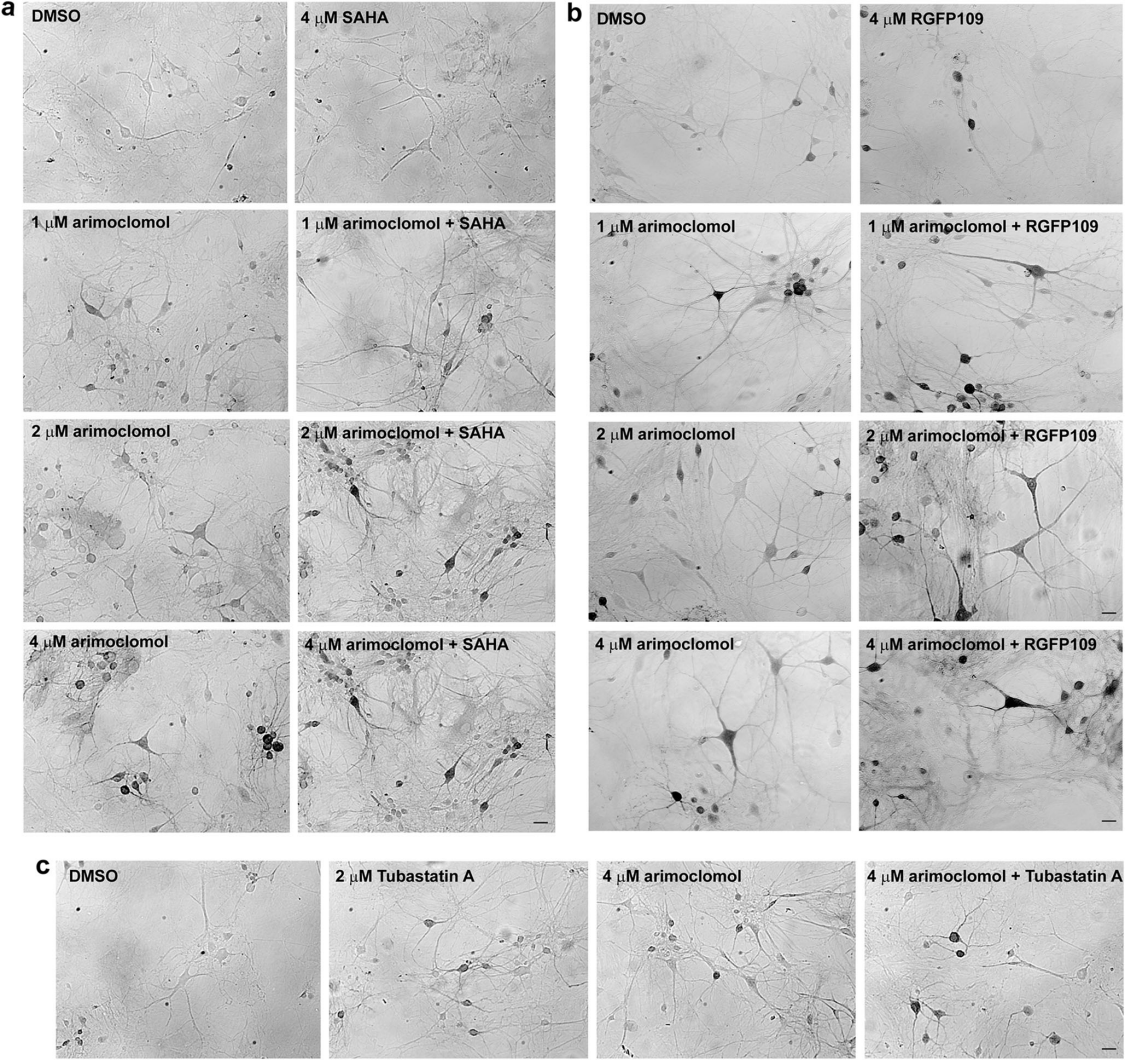
Murine spinal cord-DRG cultures were pre-treated for 30 min with vehicle (DMSO), 1–4 μM arimoclomol alone or arimoclomol in combination with different histone deacetylase inhibitors (**a** 4 μM SAHA, **b** 4 μM RGFP109, or **c** 2 μM tubastatin A), followed by heat shock at 43 °C for 30 min and recovery at 37 °C for 24 h. Cultures were fixed and immunolabeled with antibody specific for stress-inducible Hsp70 (HSPA1A), followed by HRP-conjugated antibody and development using ImmPACT DAB kit (Vector Labs SK-4105). Quantitation of the percentage of motor neurons expressing Hsp70 is presented in Fig. 6. Scale bar = 20 μm

**Fig. 6:**
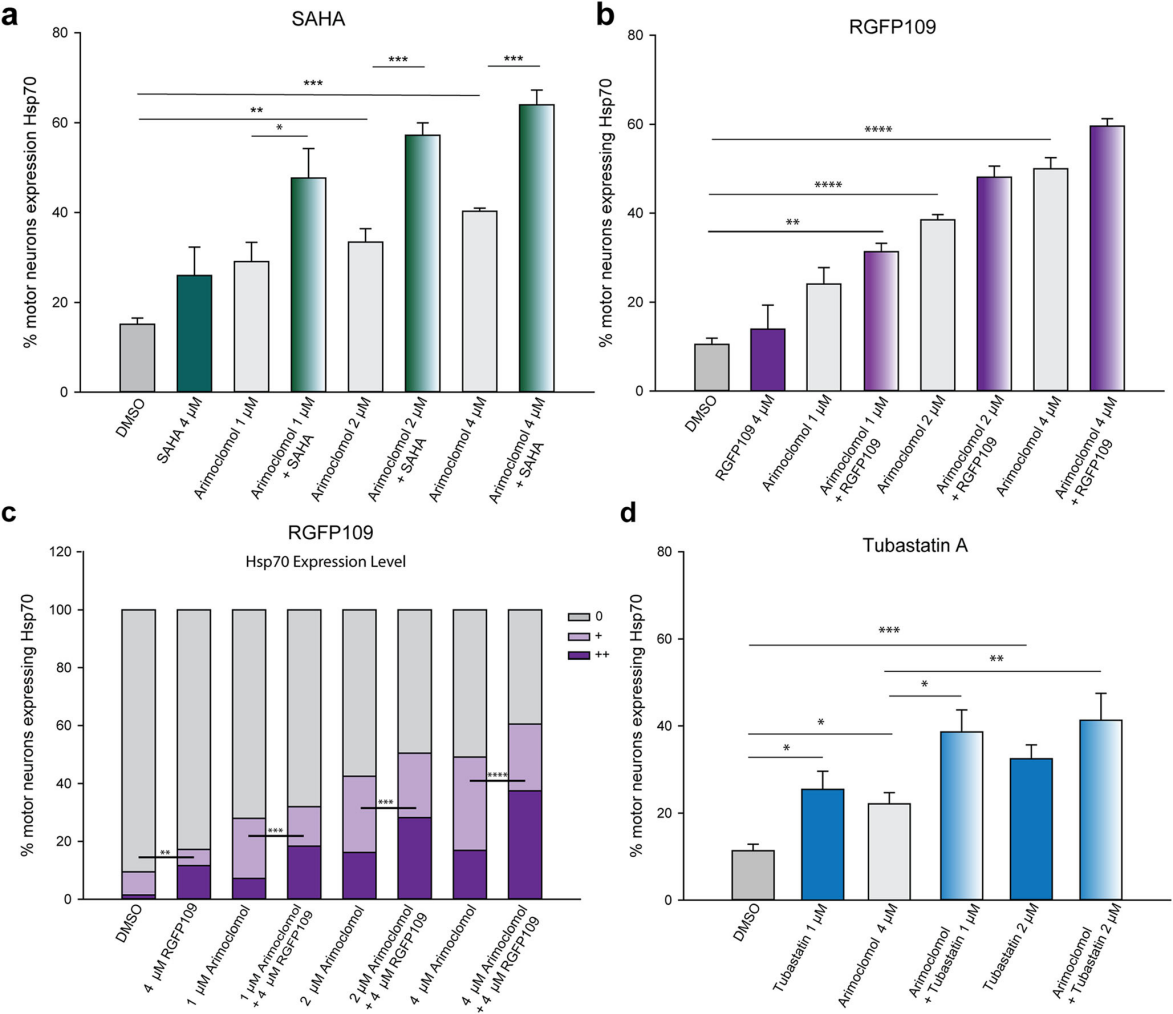
Quantitation of Hsp70 immunolabeling shown in Fig. 5. Spinal cord-DRG cultures treated with the HSP co-inducer, arimoclomol and HDAC inhibitor (**a** SAHA, **b**, **c** RGFP109, or **d** tubastatin A), alone and in combination. Cultures were heat shocked at 43 °C for 30 min and recovered at 37 °C for 24 h, then fixed and immunolabeled with antibody specific for stress-inducible Hsp70 (HSPA1A). Presence of Hsp70 labeling was scored in at least 75 motor neurons per culture. In **d**, intensity of labeling was scored as 0 (background), + (light) or ++ (strong). Presented are mean percentages ± S.E.M. of data collected from 3 cultures per condition. Statistical significance in **a**, **b**, and **d** was evaluated by one-way ANOVA and Bonferroni post hoc analysis. Analysis in **c** was by one-way MANOVA and Tukey HSD post hoc analysis: *p < 0.05 **, *p* < 0.01, ***p < 0.001, ****p < 0.0001

Arimoclomol dose-response with 24-h recovery is also shown in Fig. 5 (Hsp70 immunocytochemistry) and Fig. 6 (quantitation of the percentage of motor neurons expressing Hsp70). Compared to arimoclomol alone, Hsp70 expression was significantly higher in cultures treated with the combination of arimoclomol plus SAHA (Fig. 6a) or plus RGFP109 (Fig. 6b, c). Tubastatin A was tested at both 1 and 2 μM since some increase in histone acetylation, suggestive of inhibition of a class I HDAC can be occur with 2 μM. At both concentrations, tubastatin A had a co-inducing effect on Hsp70 expression following heat shock, similar to arimoclomol (Fig. 6d). With the combination of 1 μM tubastatin A and arimoclomol, the percentage of neurons expressing Hsp70 was slightly greater than with arimoclomol alone, but the effect was not additive.

Thus, HDAC class I inhibition potentiated Hsp70 expression by both constitutive and co-inducers of the heat shock response, at concentrations that had no effect in the absence of added stress, whereas the class IIb HDAC6 inhibitor, tubastatin A, acted as a co-inducer of Hsp70 expression in heat shocked motor neurons. Another HDAC inhibitor, phenylbutyrate, is in clinical trial for ALS; however, phenylbutyrate had no effect on Hsp70 expression in heat shocked spinal cord-DRG cultures or on the efficacy of arimoclomol or NXD30001 (Online Resource 3).

### HDAC inhibition potentiates arimoclomol-mediated enhancement of Hsp70 expression in motor neurons expressing mutant SOD1

**Fig. 7:**
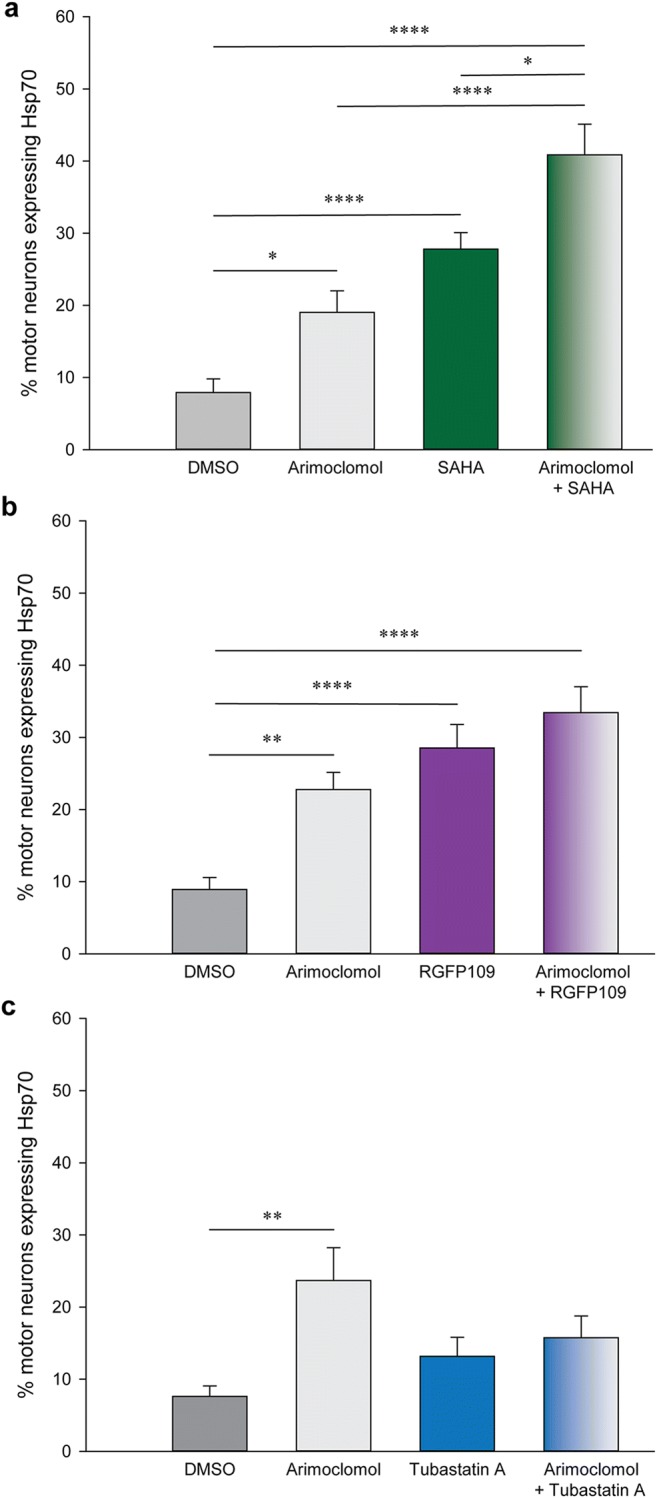
SAHA and RGFP109 act similarly to arimoclomol as co-inducers of the heat shock response in motor neurons expressing mutant SOD1 causing ALS. SOD1^G93A^ was expressed in motor neurons of dissociated spinal cord-DRG cultures by intranuclear microinjection of plasmid vector. Cultures were treated with vehicle (DMSO) or 4 μM arimoclomol, HDAC inhibitor (**a** 4 μM SAHA, **b** 4 μM RGFP109, or **c** 1 μM tubastatin A) alone and in combination. On day 3, following microinjection, cultures were fixed and labeled with antibody to stress-inducible Hsp70. Presented are mean ± SEM. Statistical significance was tested by one-way ANOVA followed by Bonferroni post hoc analysis: *p < 0.05, **p < 0.01, ***p < 0.001, ****p < 0.0001. Note: Microinjection of empty plasmid did not induce Hsp70 in any motor neurons (see text)

We next addressed whether HDAC inhibition would potentiate HSP expression by disease-causing stressors. Mutations in *SOD1* are responsible for a dominantly inherited form of ALS (Rosen et al. 1993). Mutations increase the propensity of the protein to misfold and aggregate. The HSP co-inducer, arimoclomol, delayed disease progression in SOD1^G93A^ transgenic mice (Kieran et al. 2004). We previously developed a primary culture model by expressing mutant SOD1 by intranuclear microinjection of plasmid expression vector into motor neurons of dissociated spinal cord cultures (Durham et al. 1997). Increasing expression of HSPs, by *Hsp70* gene transfer or exposure to HSP90 inhibitors including NXD30001, was protective (Batulan et al. 2006; Cha et al. 2014). Using this model, we tested the efficacy of arimoclomol to induce Hsp70 in motor neurons expressing SOD1^G93A^ and any potentiation by HDAC inhibition (Fig. 7a).

As in our previous studies (Batulan et al. 2006), expression of SOD1^G93A^ on its own was a poor inducer of Hsp70 expression. Similar to heat shock stress, less than 10% of motor neurons responded (compared to 0% of motor neurons microinjected with empty plasmid); however, this percentage was significantly increased by treatment with arimoclomol (Fig. 7a). In contrast to heat shock experiments, SAHA was as effective as arimoclomol in increasing Hsp70 in the presence of mutant SOD1 stress. However, the combination of arimoclomol and SAHA was significantly more effective than either drug alone. A similar pattern was observed with RGFP109, except the difference between RGFP109 and in combination with arimoclomol did not reach statistical significance (Fig. 7b). On the other hand, tubastatin A had no effect on Hsp70 expression in SOD1^G93A^-motor neurons, and did not potentiate the effect of arimoclomol (Fig. 7c).

### Inducible Hsp70 expression is repressed in motor neurons expressing mutant FUS

We previously reported that chromatin remodeling is altered in cultured motor neurons expressing mutant FUS linked to familial ALS (Tibshirani et al. 2015; Tibshirani et al. 2017). Toxicity of mutant FUS is associated with redistribution from the nucleus to cytoplasmic inclusions. This was accompanied by depletion of nuclear nBAF chromatin remodeling complex proteins and reduced histone acetylation.

Induction of Hsp70 was repressed in motor neurons expressing mutant FUS. Cultured motor neurons were microinjected with plasmid encoding the ALS-linked mutant, FUS^R521H^, and treated for 3 days with vehicle, SAHA (4 μM), RGFP109 (4 μM), or tubastatin A (1 μM). No neurons expressing FUS^R521H^ expressed Hsp70 were found (assessed in triplicate cultures per condition), nor was this altered by exposure to any of the HDAC inhibitors or by arimoclomol (alone or in combination with HDAC inhibitor). Similarly, expression of mutant FUS reduced the efficacy of the constitutive HSP inducer NXD30001 in motor neurons. Only 14% of motor neurons expressing FUS^R521H^ expressed Hsp70 in response to treatment with 40 nM NXD30001, compared to 60% of control neurons.

### HDAC inhibition preserves nuclear FUS in motor neurons

**Fig. 8:**
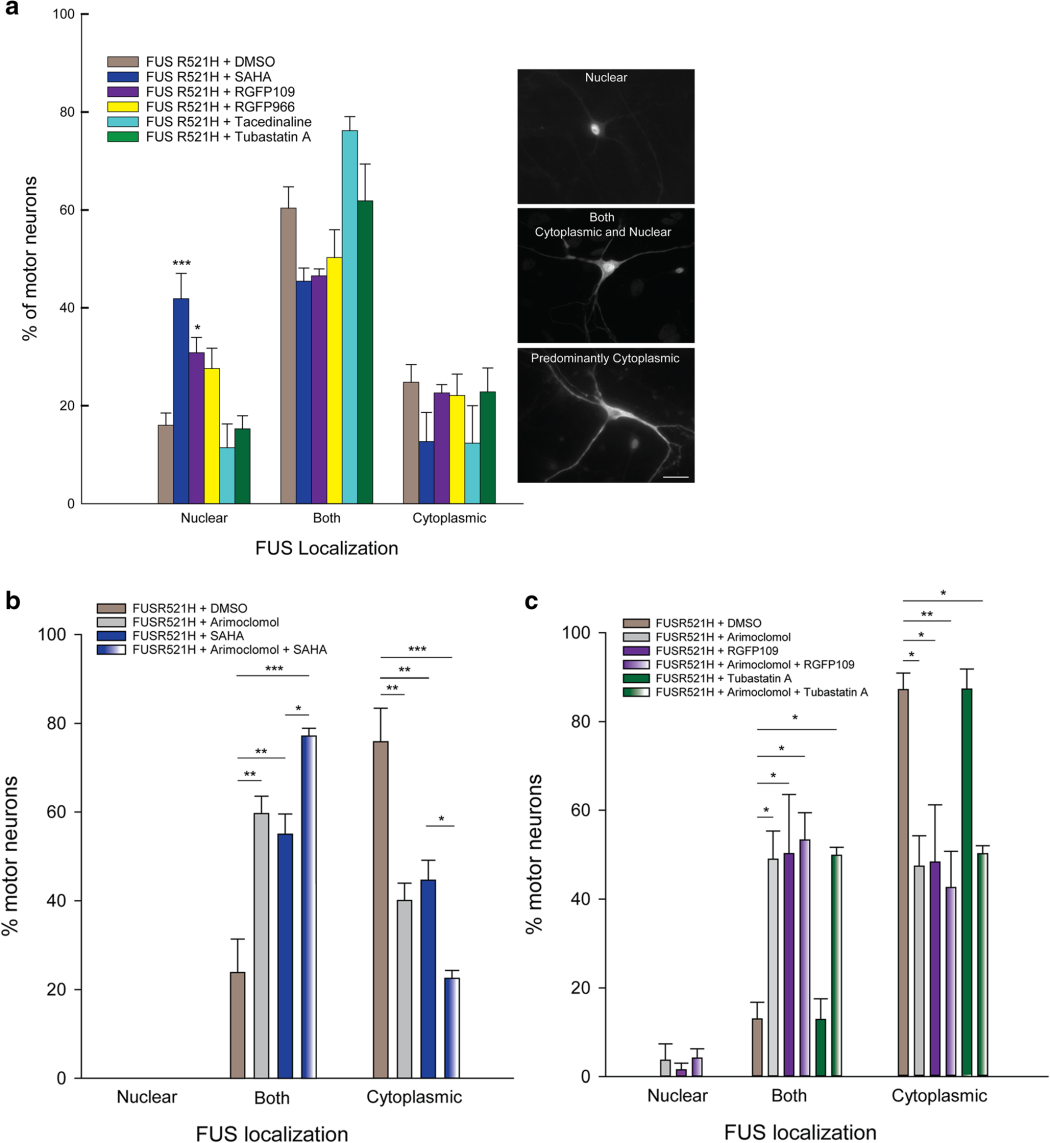
**a** The HDAC class I inhibitors, SAHA and RGFP109, retained mutant FUS in the nucleus. Flag-tagged human FUS^R521H^ was expressed in motor neurons of spinal cord-DRG cultures by intranuclear microinjection of plasmid expression vector. After 3 days of treatment with HDAC inhibitors, cultures were fixed and immunolabeled with anti-Flag M2 antibody followed by Cy2-conjugated anti-mouse IgG. The distribution of FUS was scored as nuclear, cytoplasmic or distributed in both compartments. Show are means ± SEM of counts of 20–83 motor neurons in each of 3 cultures per drug treatment group and 11 cultures vehicle (DMSO) control. Statistical analysis was performed using one-way ANOVA followed by Dunnett’s multiple comparison test relative to vehicle (with DMSO) control: *p < 0.05, **p < 0.01, ***p < 0.001. By single comparison to DMSO, RGFP966 increased the percentage of neurons with nuclear FUS with *p* value = 0.03. **b**, **c** Both arimoclomol and the HDAC inhibitors SAHA and RGFP109 retained mutant FUS in the nucleus. Human FUS^R521H^ was expressed in motor neurons of spinal cord-DRG cultures by intranuclear microinjection of plasmid expression vector. After 3 days of treatment with vehicle, arimoclomol, HDAC inhibitor (4 μM SAHA, 4 μM RGFP109, or 1 μM tubastatin A) or the combination of arimoclomol and HDAC inhibitor, cultures were fixed and immunolabeled with anti-FUS antibody followed by Cy5-conjugated anti-rabbit IgG. The distribution of FUS was scored as nuclear, cytoplasmic or distributed in both compartments. Shown are means ± SEM of counts of 17–32 motor neurons in each of 3–4 cultures per group. Statistical analysis was performed by one-way MANOVA followed by Tukey HSD post-hoc analysis: *p ≤ 0.05, **p ≤ 0.01, ***p ≤ 0.001.

Despite their failure to enhance the heat shock response in motor neurons expressing mutant FUS, class I HDAC inhibitors did promote retention of nuclear FUS. Depletion of FUS from the nucleus and accumulation in cytoplasmic inclusions is associated with multiple downstream effects including impaired nuclear functions including DNA repair, loss of nBAF chromatin remodeling complexes and attrition of intermediate and terminal dendritic branches (Tibshirani et al. 2017). Treatments that maintain nuclear FUS have the potential to prevent those downstream abnormalities. Motor neurons were microinjected with plasmid encoding mutant FUS and treated with HDAC inhibitor for 3 days. Following immunolabeling, FUS localization was scored as nuclear, cytoplasmic or distributed in both compartments (Fig. 8).

SAHA, RGP109 and RGFP966 increased the percentage of neurons with nuclear FUS compared to vehicle control. Tacedinaline and tubastatin A had no significant effect.

We next examined localization of mutant FUS in cultures treated with arimoclomol, alone and in combination with HDAC inhibitor. Interestingly, arimoclomol was as effective as SAHA (Fig. 8b) and RGFP109 (Fig. 8c) in retaining FUS in the nucleus (evident as an increase in neurons with both nuclear and cytoplasmic FUS and fewer with cytoplasmic distribution only). The combination of arimoclomol and SAHA was significantly more effective than either drug alone, whereas the combination of arimoclomol and RGFP109 had similar efficacy as each drug alone. Tubastatin A was ineffective in this experiment as well (Fig. 8c).

Thus, despite not overcoming the repression of Hsp70 expression by mutant FUS, Class I HDAC inhibitors and arimoclomol prevented the loss of nuclear FUS. This was expected of the HDAC inhibitors because of the decrease in histone acetylation correlating with aspects of mutant FUS toxicity (Tibshirani et al. 2015; Tibshirani et al. 2017), but was more surprising with arimoclomol.

### HDAC inhibitors preserve DNA repair in motor neurons expressing mutant FUS

**Fig. 9:**
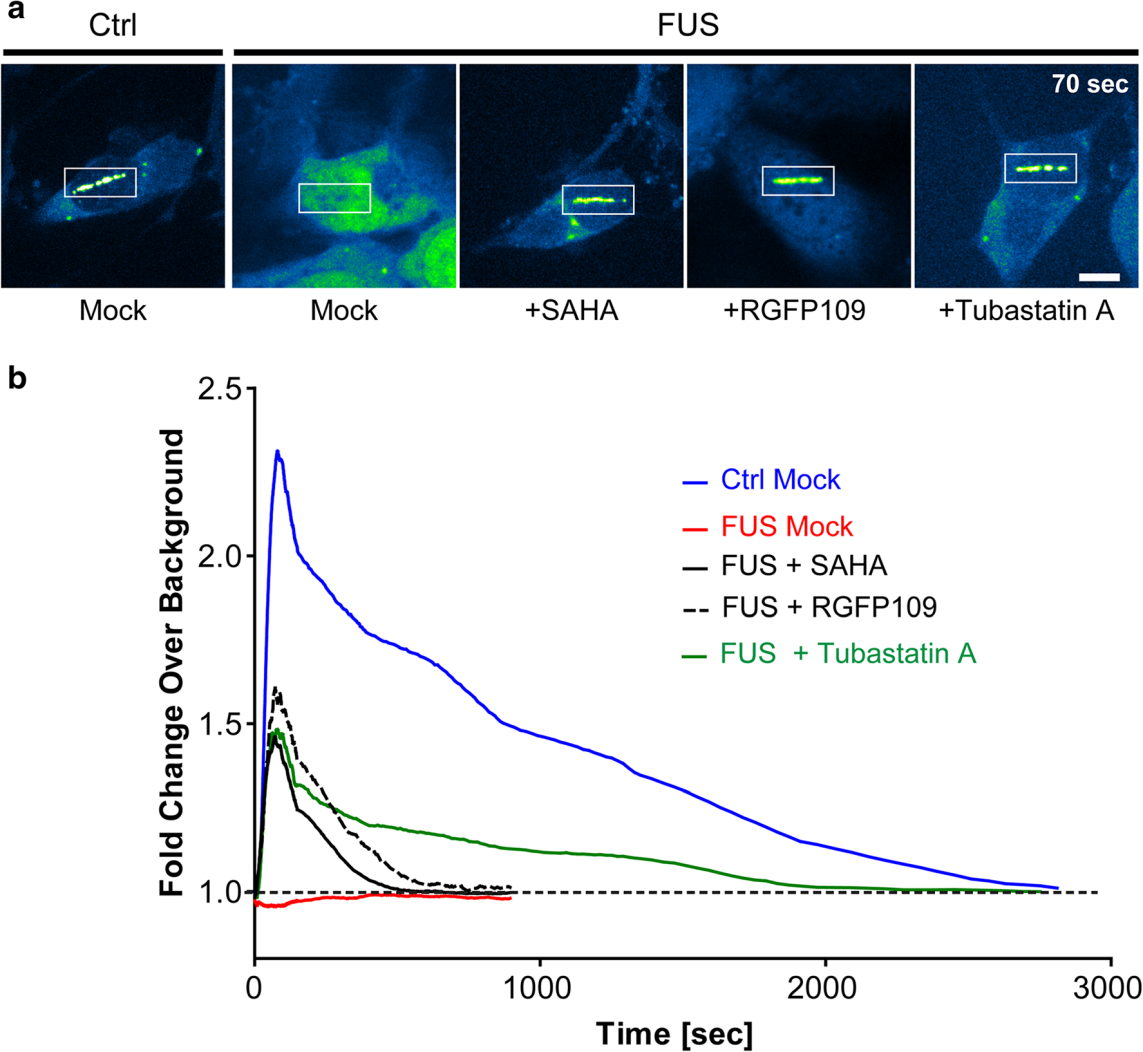
HDAC inhibitors rescued the impaired DNA damage response in motor neurons expressing an ALS-linked mutant FUS. Recruitment-withdrawal to laser irradiation sites (linear cuts) in nuclei (boxed area) of iPSC-derived spinal motor neurons expressing normal (Ctrl) or mutant P525L (FUS) FUS-GFP was imaged live at 21 DIV. **a** Mutant P525L FUS-GFP failed to be recruited to laser-irradiated DNA damage sites whilst motor neurons expressing WT FUS-GFP showed normal recruitment (Mock, compare Ctrl vs FUS). Conversely, 24-h treatment with either RGFP109 (HDAC1/3 inhibitor) or SAHA (pan HDAC inhibitor) or tubastatin A (HDAC6 inhibitor) led to a recovery of mutant FUS-GFP recruitment to laser irradiation sites. Recruitment and release of FUS-GFP at laser irradiation sites (on-off kinetics in boxed areas) was recorded with 1 fps over 15 min on a confocal live spinning disc microscope (Naumann et al. 2018). Shown is a single representative frame at 70 s of the movie. Scale bar = 10 μm. **b** Quantification of FUS-GFP at cut over time. Note the flat line of mutant FUS Mock (red line, no recruitment) and the partial, but significant rescue upon treatment with SAHA (solid black line), RGFP109 (dashed black line) or tubastatin A (green) compared to Ctrl cells (blue).

FUS plays important roles in DNA repair (Martinez-Macias et al. 2019; Mastrocola et al. 2013; Wang et al. 2013) and cells expressing mutant FUS linked to ALS accumulate DNA damage (Naumann et al. 2018; Qiu et al. 2014; Wang et al. 2013). To examine whether HDAC inhibitors would preserve DNA repair, we utilized the experimental model, in which recruitment of FUS-GFP to laser irradiation sites (linear cuts) in the nucleus (illustrated in the movie accompanying Fig. 9a), is measured in iPSC-derived motor neurons CRISPR/Cas9-engineered to express FUS^WT^-GFP or the ALS-linked mutant FUSP^525L^-GFP (Naumann et al. 2018). Whereas FUS^WT^-GFP readily accumulated at laser-irradiated, DNA damage sites (Ctrl Mock), FUS^P525L^-GFP failed to be recruited (FUS Mock). Pretreatment for 24 h with SAHA, RGFP109, or tubastatin A led to significant recovery of FUS^P525L^-GFP recruitment to laser irradiation sites (Fig. 9a, b). Thus, DNA repair in neurons expressing mutant FUS can be preserved through inhibiting activity of multiple HDACs, including HDAC6, which is better known for regulating acetylation of cytoplasmic proteins.

## Discussion

Although appealing, applying HSP-based therapy in patients has proven challenging, in part due to the high threshold for HSF1-mediated transcription in neurons (Batulan et al. 2003) and further desensitization with disease progression, which has been associated with loss of histone 4 tetra-acetylation (Labbadia et al. 2011). Acetylation has multiple influences on expression of heat shock and activity of heat shock proteins, dictating the chromatin landscape for binding of HSF1 to HSE (Guertin and Lis 2010) and regulating the activity of HSF1 and HSPs (Marinova et al. 2011; Marinova et al. 2009; Rao et al. 2012; Zelin and Freeman 2015), as well as gene expression and neuronal morphology more generally. Histone deacetylase inhibitors have neuroprotective properties on their own that are relevant to our particular interest, motor neuron disorders (Guo et al. 2017; Piepers et al. 2009; Rossaert et al. 2019; Rouaux et al. 2007; Yoo and Ko 2011), including the preservation of mutant FUS, a cause of familial ALS, in the nuclear compartment of cultured motor neurons as reported in the present study.

We evaluated the ability of different classes of histone deacetylase inhibitors to enable the heat shock response in motor neurons, both alone and in combination with drugs that induce HSP expression constitutively or magnify induction in stressed cells (co-inducers). The Hsp70, HSPA1A, was used as the classic marker of stress-induced HSP expression in the following experimental paradigms in dissociated cultures of murine spinal cord-DRG: Constitutive induction of Hsp70 by an HSP90 inhibitor, thermal stress, and expression of mutant proteins (SOD1 and FUS) responsible for familial forms of ALS as models of proteotoxic stress linked to neurodegenerative disease. Heat shock and the ALS models also served as initiating stresses to evaluate the HSP co-inducer, arimoclomol, which is in clinical trial for ALS and inclusion body myositis and exhibits a favorable safety profile (Lanka et al. 2009), as well as to determine if efficacy would be increased by combination with an HDAC inhibitor. The results are summarized in Table 2.

In reference to HSP90 inhibition, heat shock and expression of mutant SOD1, although there were some differences according to the stress paradigms, the premise that HDAC inhibition would enhance the efficacy of HSP inducers holds true. Class I HDACs were implicated since the Class I HDAC1/3 inhibitor, RGFP109 produced similar results as the pan HDAC inhibitor SAHA, whereas effects of the class IIb inhibitor, tubastatin A were inconsistent with different stress paradigms (see below). HDAC1 is implicated since the HDAC1 inhibitor, tacedinaline, was effective in potentiating NXD30001-induced Hsp70 expression in motor neurons, but the HDAC3 inhibitor, RGFP966, was not. It is also possible that combined inhibition of multiple class I HDACs is important, as appears to be the case in models of Friedreich’s Ataxia (Chutake et al. 2016).

The class IIb HDAC6 inhibitor, tubastatin A, showed enhanced heat shock-induced Hsp70, alone and when combined with arimoclomol, and increased neuronal, but not non-neuronal, Hsp70 induced by the HSP90 inhibitor, NXD30001, yet it was ineffective in the ALS models with respect to Hsp70 induction. HDAC6 is an important regulator of HSF1 activation, but this action depends on the stress, HSF1 being activated by HDAC6-dependent and independent mechanisms (Boyault et al. 2007; Pernet et al. 2014). In unstressed cells, HSF1 is complexed with HDAC6, p97/VCP, HSP90, and other HSPs and must be released to interact with HSE in *HSP* gene promoters. Heat directly disrupts the HSP90-HSF1 complex, whereas with proteotoxic stress, as occurs with proteasome inhibition, HSF1 activation is driven by HDAC6 binding to polyubiquitinated proteins, resulting in the p97/VCP-dependent segregation of HSF1-HSP90 complexes. As the amount of ubiquitinated protein diminishes, the inactive complexes can reform, thereby regulating the duration of HSF1 activation in a manner proportional to the intensity of the proteotoxic stress (Pernet et al. 2014). This mechanism is likely to operate in neurons expressing mutant SOD1, which misfolds and aggregates and inhibits the proteasome (Kabashi et al. 2012). Consistent with our data, tubastatin A reduced proteasome inhibitor-driven upregulation of HSP70 and autophagic flux in cultured oligodendrocytes (Leyk et al. 2015). The differential involvement of HDAC6 in thermal versus proteotoxic stress could help to explain why tubastatin A had different effects in this study depending on the cellular stress model. It should also be noted that HSF1 is not the only transcription factor activating HSP gene transcription. Proteasome inhibition can also act through the transcription factor HSF2 (Batulan et al. 2003) and the Hsp70 promoter also contains other potentially relevant elements including SP1 (Taylor et al. 2007). The phosphatidylinositol 3-kinase/Akt pathway and Sp1 have been implicated in Hsp70 induction by HDAC inhibitors (Marinova et al. 2009).

Interestingly, SAHA and RGFP109 acted on their own as HSP co-inducers in the SOD1 model, but not with HSP90 inhibition or heat shock—the opposite of tubastatin A. SAHA and RGFP109 exhibited similar activity as arimoclomol with respect to Hsp70 induction in motor neurons expressing mutant SOD1. Arimoclomol’s effect was qualitatively consistent with other studies in cultured motor neurons exposed to staurosporin or hydrogen peroxide (Kalmar and Greensmith 2009) and with experiments in SOD1^G93A^ transgenic mice, in which treatment with arimoclomol restored Hsp70 levels in spinal motor neurons (Kieran et al. 2004). With this stress paradigm, the combination of SAHA and arimoclomol was significantly better than either drug alone, supporting our hypothesis of improved efficacy with combined treatment.

What mechanism might account for potentiation of NXD30001 by tubastatin A? HDAC6 acetylation of non-histone proteins, including HSP90 and HSF1, has complicated effects on HSP expression (Marinova et al. 2011; Marinova et al. 2009; Rao et al. 2012; Zelin and Freeman 2015). HDAC6 is a client protein and deacetylase of HSP90. Increased acetylation disrupts function of HSP90 and therefore its clients (Bali et al. 2005; Rao et al. 2008); thus, inhibiting deacetylation of HSP90 by tubastatin A and inhibiting HSP90’s active site by NXD30001 could both facilitate disruption of complexes and release of HSF1, the combined effect being greater that individual drug.

A very different pattern was observed with expression of mutant FUS linked to familial ALS. A number of genes linked to ALS encode RNA binding proteins, including FUS (Kwiatkowski Jr. et al. 2009; Vance et al. 2009). In our primary culture model (Tradewell et al. 2012) histone acetylation is impaired in motor neurons expressing mutant FUS, but preserved by treatment with SAHA (Tibshirani et al. 2015). Expression of mutant FUS not only failed to induce Hsp70 in motor neurons, but impaired the efficacy of HSP-inducing drugs, both the HSP90 inhibitor, NXD30001, and the HSP co-inducer, arimoclomol. Thus, mutant FUS suppresses the heat shock response, an effect that could compromise cellular defense mechanisms and contribute to motor neuron dysfunction and loss. The actual mechanism of this suppression is not known, but could relate to epigenetic changes impairing chromatin accessibility at heat shock gene promoters (Budzynski et al. 2017; Chen et al. 2002; Tibshirani et al. 2017). Regardless, it was relevant to determine whether different classes of HDAC inhibitors could restore stress-induced expression of HSPs and enable HSP inducing drugs. Neither SAHA nor RGFP109 nor tubastatin A were effective in inducing Hsp70; however, SAHA and RGFP109 both significantly retained mutant FUS in the nuclear compartment. The ineffectiveness of the specific class IIb (HDAC6) inhibitor, tubastatin A, points to inhibition of class I HDACs underlying the effect of SAHA and RGFP109. The class I HDAC1 inhibitor, tacedinaline, also was completely ineffective in this assay, but the improvement in nuclear retention of FUS by the class I HDAC3 inhibitor, RGFP966, although only reaching statistical significance in single comparison to DMSO (*p* = 0.03), is consistent with HDAC3 inhibition contributing to the activity of the HDAC1/3 inhibitor, RGFP109, and SAHA. Of note for comparison. RGFP966 failed to potentiate NXD30001-induced Hsp70.

Given the many nuclear functions of FUS, retention of nuclear FUS would have multiple downstream effects to maintain neuronal function. In this model, depletion of nuclear FUS was associated with loss of histone acetylation (specifically H3K9/K14), a crucial transcriptional regulator (Tibshirani et al. 2015), loss of nBAF chromatin remodeling complexes and dendritic attrition (Tibshirani et al. 2017), effects that are prevented by SAHA. FUS plays an important role in the DNA damage response by being recruited to sites of breakage in a PAR-dependent manner and this recruitment is inhibited in cells expressing mutant FUS (Martinez-Macias et al. 2019; Naumann et al. 2018). In iPSC-derived motor neurons engineered to express FUS^P525L^-GFP. SAHA and RGFP109 significantly restored recruitment of FUS^P525L^-GFP to sites of laser-induced DNA damage, although not to control levels. Surprisingly, tubastatin A also showed efficacy in this assay, a result that is neither expected nor explained given its specificity for HDAC6, a cytoplasmic enzyme, at the concentration used. The normal function of FUS includes recruiting HDAC1 to sites of induced DNA damage, and this process is perturbed in cells expressing mutant FUS (Wang et al. 2013); however, a role for HDAC6 is not evident.

A particularly interesting finding of this study was that arimoclomol, although not inducing Hsp70 in motor neurons expressing mutant FUS, was as effective as SAHA and RGFP109 in retaining nuclear FUS. Given the high threshold for activation of the heat shock response in certain types of neurons, it has been suspected that this drug has other neuroprotective mechanisms. Since this study and others have shown HSP-inducing properties of HDAC inhibitors (Budzynski et al. 2017; Chen et al. 2002; Ren et al. 2004; Tao et al. 2004; Zhao et al. 2005). we questioned whether arimoclomol has HDAC inhibitory activity. Activity of HDAC class I enzymes was not significantly inhibited in HeLa cell nuclear extracts (supplied with the HDAC activity assay kit) or whole cells homogenate of spinal cord-DRG cultures exposed to arimoclomol at the concentration used in this study (4 μM) (Online Resource 4a,b). As expected, RGFP109 did inhibit class I HDAC activity (by 46% in spinal cord-DRG cultures relative to vehicle control—Online Resource 4b). Budzynski et al. recently reported that treatment with another HSP co-inducer, BPG-15, resulted in reduced HDAC activity in cultured mouse embryo fibroblasts, particularly in isolated nuclei, although activity of purified HDAC enzymes (1, 4, 6, and 10) was not compromised. They concluded that an indirect effect of BPG-15, such as on multiprotein HDAC assemblies influencing HDAC activity, relates to increased chromatin accessibility at heat shock genes and enhancement of the heat shock response. Given that acetylation impacts multiple processes regulating the heat shock response, more work will be needed to understand how these data fit into the mechanism of action of HSP co-inducers. It has long been thought that arimoclomol has neuroprotective properties separate from HSPs, but the data do not support direct HDAC inhibition being this mechanism (no significant effect on HDAC class I activity—Online Resource 4).

With respect to FUS-induced proteotoxicity, histone acetylation is reduced in experimental models and prevented by HDAC inhibitors, accompanied by retention of nuclear FUS (Rossaert et al. 2019; Tibshirani et al. 2015). The finding in the present study that arimoclomol had activity similar to RGFP109 and SAHA in preserving nuclear FUS would argue against a small indirect effect on HDAC activity being responsible.

In terms of how compounds with more substantial HDAC inhibitory activity enhance the heat shock response, the mechanisms regulating HSP expression are complex, as is the involvement of HDACs in those processes. The HSP enhancing properties of a particular HDAC may not be completely predictable by knowing its substrate specificity and given that regulatory mechanisms vary with the type and level of the stress, a potential drug would need to be evaluated in an experimental paradigm appropriate for the circumstance of use. For example, the pan HDAC inhibitor SAHA was most effective overall in the assays tested, but this property did not extend to another nonspecific HDAC inhibitor, phenylbutyrate. However, overall, the class I HDACs are favored for their activity as HSP co-inducers as well as epigenetic modifiers, conditions that are common in neurodegenerative disorders.

In summary, histone acetylation and chromatin remodeling complexes play crucial and interdependent roles in regulating gene expression for neuronal form and function and for cellular defense. The data support the idea of combining an HDAC inhibitor with an HSP-inducer, not only to improve, but to prolong efficacy. This combination could also ameliorate epigenetic changes and more widespread disruption of gene expression in ALS, either directly by maintaining histone acetylation, or indirectly by maintaining protein quality and neuronal homeostasis.

## Electronic supplementary material

ESM 1 (PDF 922 kb)

## References

[CR1] Bali P, Pranpat M, Bradner J, Balasis M, Fiskus W, Guo F, Rocha K, Kumaraswamy S, Boyapalle S, Atadja P, Seto E, Bhalla K (2005). Inhibition of histone deacetylase 6 acetylates and disrupts the chaperone function of heat shock protein 90: a novel basis for antileukemia activity of histone deacetylase inhibitors. J Biol Chem.

[CR2] Batulan Z, Nalbantoglu J, Durham HD (2005). Nonsteroidal anti-inflammatory drugs differentially affect the heat shock response in cultured spinal cord cells. Cell Stress Chaperones.

[CR3] Batulan Zarah, Shinder Gayle A., Minotti Sandra, He Bei Ping, Doroudchi Mohammad M., Nalbantoglu Josephine, Strong Michael J., Durham Heather D. (2003). High Threshold for Induction of the Stress Response in Motor Neurons Is Associated with Failure to Activate HSF1. The Journal of Neuroscience.

[CR4] Batulan Z, Taylor DM, Aarons RJ, Minotti S, Doroudchi MM, Nalbantoglu J, Durham HD (2006). Induction of multiple heat shock proteins and neuroprotection in a primary culture model of familial amyotrophic lateral sclerosis. Neurobiol Dis.

[CR5] Benoy V (2018). HDAC6 is a therapeutic target in mutant GARS-induced Charcot-Marie-Tooth disease. Brain.

[CR6] Boyault C (2007). HDAC6 controls major cell response pathways to cytotoxic accumulation of protein aggregates. Genes Dev.

[CR7] Budzynski MA, Crul T, Himanen SV, Toth N, Otvos F, Sistonen L, Vigh L (2017). Chaperone co-inducer BGP-15 inhibits histone deacetylases and enhances the heat shock response through increased chromatin accessibility. Cell Stress Chaperones.

[CR8] Budzynski MA, Puustinen MC, Joutsen J, Sistonen L (2015). Uncoupling stress-inducible phosphorylation of heat shock factor 1 from its activation. Mol Cell Biol.

[CR9] Calderwood SK, Xie Y, Wang X, Khaleque MA, Chou SD, Murshid A, Prince T, Zhang Y (2010). Signal transduction pathways leading to heat shock transcription. Sign Transduct Insights.

[CR10] Cha JR (2014). A novel small molecule HSP90 inhibitor, NXD30001, differentially induces heat shock proteins in nervous tissue in culture and in vivo. Cell Stress Chaperones.

[CR11] Chen T, Sun H, Lu J, Zhao Y, Tao D, Li X, Huang B (2002). Histone acetylation is involved in hsp70 gene transcription regulation in Drosophila melanogaster. Arch Biochem Biophys.

[CR12] Chutake Yogesh K., Lam Christina C., Costello Whitney N., Anderson Michael P., Bidichandani Sanjay I. (2016). Reversal of epigenetic promoter silencing in Friedreich ataxia by a class I histone deacetylase inhibitor. Nucleic Acids Research.

[CR13] Cudkowicz ME (2009). Phase 2 study of sodium phenylbutyrate in ALS. Amyotroph Lateral Scler.

[CR14] d'Ydewalle C (2011). HDAC6 inhibitors reverse axonal loss in a mouse model of mutant HSPB1-induced Charcot-Marie-Tooth disease. Nat Med.

[CR15] Dayalan Naidu S, Dinkova-Kostova AT (2017). Regulation of the mammalian heat shock factor 1. FEBS J.

[CR16] Durham HD, Roy J, Dong L, Figlewicz DA (1997). Aggregation of mutant Cu/Zn superoxide dismutase proteins in a culture model of ALS. J Neuropathol Exp Neurol.

[CR17] Guertin MJ, Lis JT (2010). Chromatin landscape dictates HSF binding to target DNA elements. PLoS Genet.

[CR18] Guo W (2017). HDAC6 inhibition reverses axonal transport defects in motor neurons derived from FUS-ALS patients. Nat Commun.

[CR19] Janssen C, Schmalbach S, Boeselt S, Sarlette A, Dengler R, Petri S (2010). Differential histone deacetylase mRNA expression patterns in amyotrophic lateral sclerosis. J Neuropathol Exp Neurol.

[CR20] Joutsen Jenny, Sistonen Lea (2018). Tailoring of Proteostasis Networks with Heat Shock Factors. Cold Spring Harbor Perspectives in Biology.

[CR21] Kabashi E, Agar JN, Strong MJ, Durham HD (2012). Impaired proteasome function in sporadic amyotrophic lateral sclerosis. Amyotroph Lateral Scler.

[CR22] Kalmar B, Greensmith L (2009) Activation of the heat shock response in a primary cellular model of motoneuron neurodegeneration-evidence for neuroprotective and neurotoxic effects. Cell Mol Biol Lett. 10.2478/s11658-009-0002-8 10.2478/s11658-009-0002-8 PMC6275696 19183864

[CR23] Kieran D, Kalmar B, Dick JR, Riddoch-Contreras J, Burnstock G, Greensmith L (2004). Treatment with arimoclomol, a coinducer of heat shock proteins, delays disease progression in ALS mice. Nat Med.

[CR24] Kijima T (2018). HSP90 inhibitors disrupt a transient HSP90-HSF1 interaction and identify a noncanonical model of HSP90-mediated HSF1 regulation. Sci Rep.

[CR25] Kwiatkowski TJ (2009). Mutations in the FUS/TLS gene on chromosome 16 cause familial amyotrophic lateral sclerosis. Science.

[CR26] Labbadia J (2011). Altered chromatin architecture underlies progressive impairment of the heat shock response in mouse models of Huntington disease. J Clin Invest.

[CR27] Lanka V, Wieland S, Barber J, Cudkowicz M (2009). Arimoclomol: a potential therapy under development for ALS. Expert Opin Investig Drugs.

[CR28] Leyk J, Goldbaum O, Noack M, Richter-Landsberg C (2015). Inhibition of HDAC6 modifies tau inclusion body formation and impairs autophagic clearance. J Mol Neurosci : MN.

[CR29] Li J, Labbadia J, Morimoto RI (2017). Rethinking HSF1 in stress, development, and organismal health. Trends Cell Biol.

[CR30] Liu D (2013). Proteomic analysis reveals differentially regulated protein acetylation in human amyotrophic lateral sclerosis spinal cord. PLoS One.

[CR31] Manzerra P, Brown IR (1992). Expression of heat shock genes (hsp70) in the rabbit spinal cord: localization of constitutive and hyperthermia-inducible mRNA species. J Neurosci Res.

[CR32] Marinova Z, Leng Y, Leeds P, Chuang DM (2011). Histone deacetylase inhibition alters histone methylation associated with heat shock protein 70 promoter modifications in astrocytes and neurons. Neuropharmacology.

[CR33] Marinova Z (2009). Valproic acid induces functional heat-shock protein 70 via class I histone deacetylase inhibition in cortical neurons: a potential role of Sp1 acetylation. J Neurochem.

[CR34] Martinez-Macias Maria Isabel, Moore Duncan AQ, Green Ryan L, Gomez-Herreros Fernando, Naumann Marcel, Hermann Andreas, Van Damme Philip, Hafezparast Majid, Caldecott Keith W (2019). FUS (fused in sarcoma) is a component of the cellular response to topoisomerase I–induced DNA breakage and transcriptional stress. Life Science Alliance.

[CR35] Mastrocola AS, Kim SH, Trinh AT, Rodenkirch LA, Tibbetts RS (2013). The RNA-binding protein fused in sarcoma (FUS) functions downstream of poly(ADP-ribose) polymerase (PARP) in response to DNA damage. J Biol Chem.

[CR36] Morimoto RI (1998). Regulation of the heat shock transcriptional response: cross talk between a family of heat shock factors, molecular chaperones, and negative regulators. Genes Dev.

[CR37] Naumann M, Pal A, Goswami A, Lojewski X, Japtok J, Vehlow A, Naujock M, Günther R, Jin M, Stanslowsky N, Reinhardt P, Sterneckert J, Frickenhaus M, Pan-Montojo F, Storkebaum E, Poser I, Freischmidt A, Weishaupt JH, Holzmann K, Troost D, Ludolph AC, Boeckers TM, Liebau S, Petri S, Cordes N, Hyman AA, Wegner F, Grill SW, Weis J, Storch A, Hermann A (2018). Impaired DNA damage response signaling by FUS-NLS mutations leads to neurodegeneration and FUS aggregate formation. Nat Commun.

[CR38] Pal A, Glass H, Naumann M, Kreiter N, Japtok J, Sczech R, Hermann A (2018). High content organelle trafficking enables disease state profiling as powerful tool for disease modelling. Sci Data.

[CR39] Pernet L, Faure V, Gilquin B, Dufour-Guerin S, Khochbin S, Vourc'h C (2014). HDAC6-ubiquitin interaction controls the duration of HSF1 activation after heat shock. Mol Biol Cell.

[CR40] Piepers S (2009). Randomized sequential trial of valproic acid in amyotrophic lateral sclerosis. Ann Neurol.

[CR41] Qiu H (2014). ALS-associated mutation FUS-R521C causes DNA damage and RNA splicing defects. J Clin Invest.

[CR42] Rao R, Fiskus W, Ganguly S, Kambhampati S, Bhalla KN (2012). HDAC inhibitors and chaperone function. Adv Cancer Res.

[CR43] Rao R (2008). HDAC6 inhibition enhances 17-AAG--mediated abrogation of hsp90 chaperone function in human leukemia cells. Blood.

[CR44] Raychaudhuri S (2014). Interplay of acetyltransferase EP300 and the proteasome system in regulating heat shock transcription factor 1. Cell.

[CR45] Reinhardt P, Glatza M, Hemmer K, Tsytsyura Y, Thiel CS, Höing S, Moritz S, Parga JA, Wagner L, Bruder JM, Wu G, Schmid B, Röpke A, Klingauf J, Schwamborn JC, Gasser T, Schöler HR, Sterneckert J (2013). Derivation and expansion using only small molecules of human neural progenitors for neurodegenerative disease modeling. PLoS One.

[CR46] Ren M, Leng Y, Jeong M, Leeds PR, Chuang DM (2004). Valproic acid reduces brain damage induced by transient focal cerebral ischemia in rats: potential roles of histone deacetylase inhibition and heat shock protein induction. J Neurochem.

[CR47] Rosen DR (1993). Mutations in Cu/Zn superoxide dismutase gene are associated with familial amyotrophic lateral sclerosis. Nature.

[CR48] Rossaert E, Pollari E, Jaspers T, van Helleputte L, Jarpe M, van Damme P, de Bock K, Moisse M, van den Bosch L (2019). Restoration of histone acetylation ameliorates disease and metabolic abnormalities in a FUS mouse model. Acta Neuropathol Commun.

[CR49] Rouaux C, Jokic N, Mbebi C, Boutillier S, Loeffler JP, Boutillier AL (2003). Critical loss of CBP/p300 histone acetylase activity by caspase-6 during neurodegeneration. EMBO J.

[CR50] Rouaux C (2007). Sodium valproate exerts neuroprotective effects in vivo through CREB-binding protein-dependent mechanisms but does not improve survival in an amyotrophic lateral sclerosis mouse model. J Neurosci.

[CR51] Roy J, Minotti S, Dong L, Figlewicz DA, Durham HD (1998). Glutamate potentiates the toxicity of mutant Cu/Zn-superoxide dismutase in motor neurons by postsynaptic calcium-dependent mechanisms. J Neurosci.

[CR52] Ryu H (2005). Sodium phenylbutyrate prolongs survival and regulates expression of anti-apoptotic genes in transgenic amyotrophic lateral sclerosis mice. J Neurochem.

[CR53] Shen W (2007). Solution structure of human Brg1 bromodomain and its specific binding to acetylated histone tails. Biochemistry.

[CR54] Shivaswamy S, Iyer VR (2008). Stress-dependent dynamics of global chromatin remodeling in yeast: dual role for SWI/SNF in the heat shock stress response. Mol Cell Biol.

[CR55] Sullivan EK, Weirich CS, Guyon JR, Sif S, Kingston RE (2001). Transcriptional activation domains of human heat shock factor 1 recruit human SWI/SNF. Mol Cell Biol.

[CR56] Tao D, Lu J, Sun H, Zhao YM, Yuan ZG, Li XX, Huang BQ (2004). Trichostatin A extends the lifespan of Drosophila melanogaster by elevating hsp22 expression. Acta Biochim Biophys Sin Shanghai.

[CR57] Taylor DM, De KP, Minotti S, Durham HD (2007). Manipulation of protein kinases reveals different mechanisms for upregulation of heat shock proteins in motor neurons and non-neuronal cells. Mol Cell Neurosci.

[CR58] Tibshirani M (2015). Cytoplasmic sequestration of FUS/TLS associated with ALS alters histone marks through loss of nuclear protein arginine methyltransferase 1. Hum Mol Genet.

[CR59] Tibshirani M (2017). Dysregulation of chromatin remodelling complexes in amyotrophic lateral. Sclerosis Hum Mol Genet.

[CR60] Tradewell ML, Yu Z, Tibshirani M, Boulanger M-C, Durham HD, Richard S (2012). Arginine methylation by PRMT1 regulates nuclear-cytoplasmic localization and toxicity of FUS/TLS harbouring ALS-linked mutations. Hum Mol Genet.

[CR61] Vance C (2009). Mutations in FUS, an RNA processing protein, cause familial amyotrophic lateral sclerosis type 6. Science.

[CR62] Vera M, Pani B, Griffiths LA, Muchardt C, Abbott CM, Singer RH, Nudler E (2014). The translation elongation factor eEF1A1 couples transcription to translation during heat shock response. Elife.

[CR63] Vogel-Ciernia A, Wood MA (2013). Neuron-specific chromatin remodeling: a missing link in epigenetic mechanisms underlying synaptic plasticity, memory, and intellectual disability disorders. Neuropharmacology.

[CR64] Wang WY (2013). Interaction of FUS and HDAC1 regulates DNA damage response and repair in neurons. Nat Neurosci.

[CR65] Westerheide SD, Anckar J, Stevens SM, Sistonen L, Morimoto RI (2009). Stress-inducible regulation of heat shock factor 1 by the deacetylase SIRT1. Science.

[CR66] Wu JI, Lessard J, Olave IA, Qiu Z, Ghosh A, Graef IA, Crabtree GR (2007). Regulation of dendritic development by neuron-specific chromatin remodeling complexes. Neuron.

[CR67] Yoo YE, Ko CP (2011). Treatment with trichostatin a initiated after disease onset delays disease progression and increases survival in a mouse model of amyotrophic lateral sclerosis. Exp Neurol.

[CR68] Zelin E, Freeman BC (2015). Lysine deacetylases regulate the heat shock response including the age-associated impairment of HSF1. J Mol Biol.

[CR69] Zhao Y, Sun H, Lu J, Li X, Chen X, Tao D, Huang W, Huang B (2005). Lifespan extension and elevated hsp gene expression in Drosophila caused by histone deacetylase inhibitors. J Exp Biol.

