# Supplementary material for: Depending on the stress, histone deacetylase inhibitors act as heat shock protein co-inducers in motor neurons and potentiate arimoclomol, exerting neuroprotection through multiple mechanisms in ALS models
Source: Cell Stress Chaperones. 2020 Jan 3;25(1):173–91. doi: 10.1007/s12192-019-01064-1 (PMC6985055; doi:10.1007/s12192-019-01064-1)
Supplement: Supplementary file 1 — (PDF 922 kb) [file 12192_2019_1064_MOESM1_ESM.pdf]

Online Resources

Depending on the stress, histone deacetylase inhibitors act as heat shock protein co-inducers in motor neurons and potentiate arimoclomol, exerting neuroprotection through multiple mechanisms in ALS models

Rachel Kuta, Nancy Larochelle, Mario Fernandez, Arun Pal, Sandra Minotti, Michael Tibshirani, Kyle St. Louis, Benoit J. Gentil, Josephine N. Nalbantoglu, Andreas Hermann, Heather D. Durham

Corresponding author: Heather D. Durham, Department of Neurology and Neurosurgery and Montreal Neurological Institute, McGill University **heather.durham@mcgill.ca**

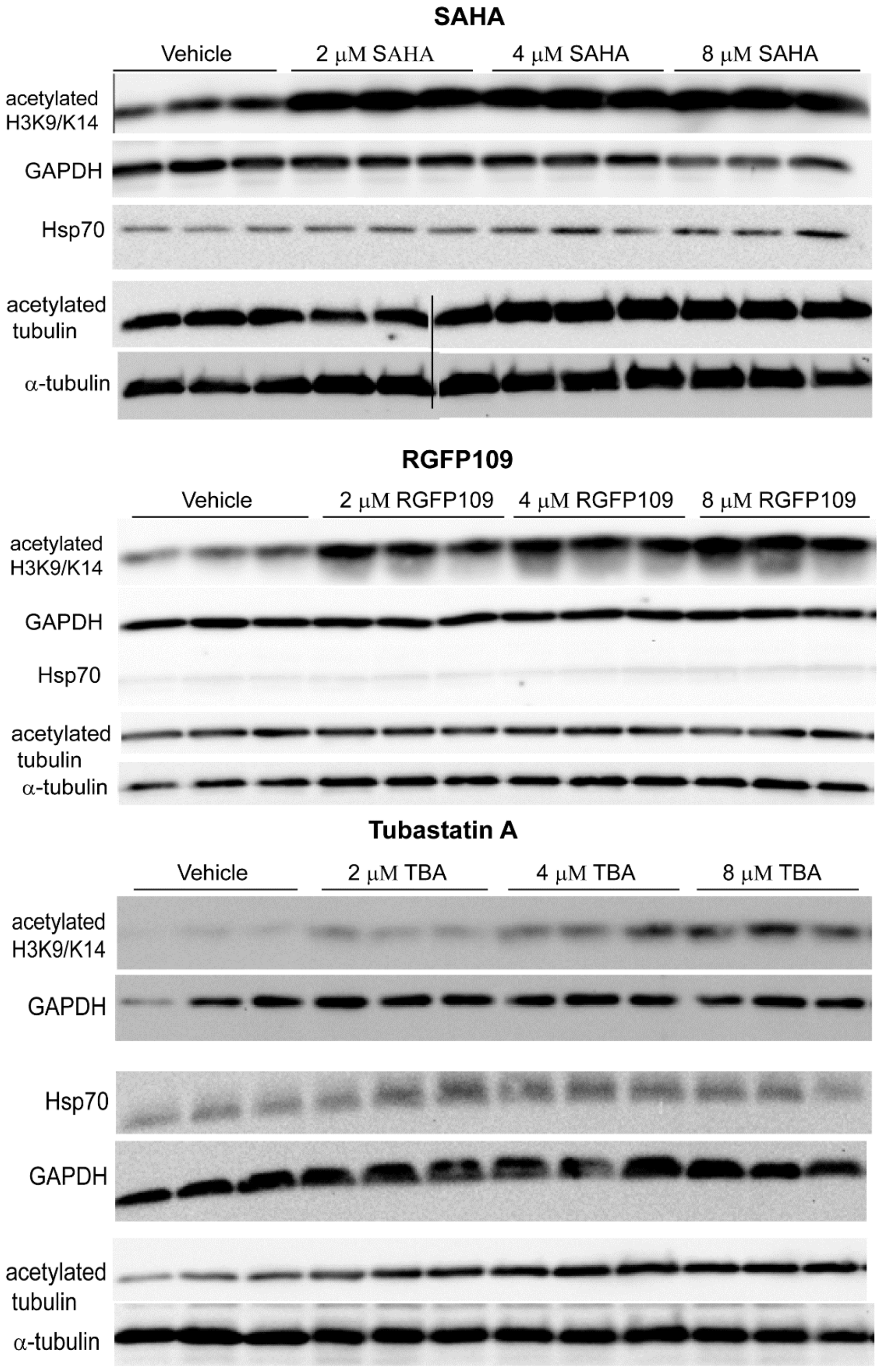

Online Resource 1 Original Western blot data supporting the graphs of dose-response for HDAC inhibitors on levels of acetylated H3K9/K14 and acetylated tubulin (Fig. 1) and Hsp70 (Fig. 2) in cultured spinal cord-DRG cultures. Labeling of acetylated H3K9/K14 and Hsp70 was expressed relative to GAPDH and acetylated tubulin was calculated relative to total α-tubulin. Procedures for Western blotting and antibodies used are described in the Materials and Methods. Note, the vertical line in the blots for tubulin in the SAHA panel indicates removal of a lane without sample in order to align with the other blots.

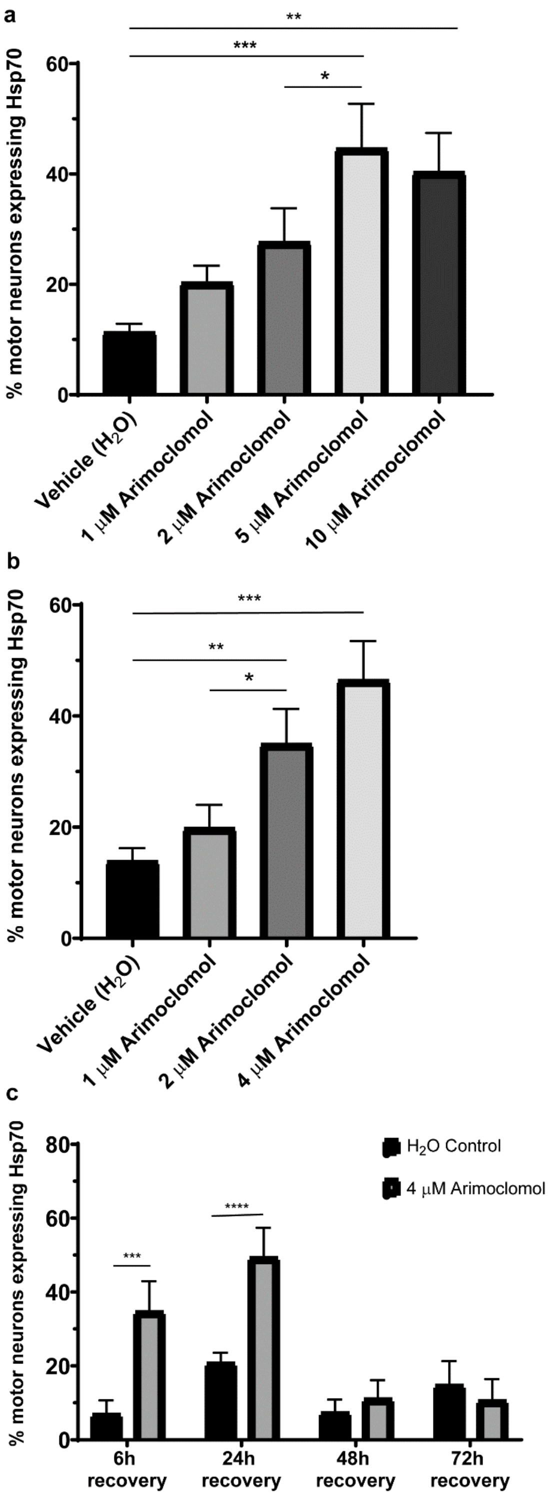

Online Resource 2 Treatment with the HSP coinducer, arimoclomol, induced expression of Hsp70 in heat shocked motor neurons in a dose-dependent manner.

a,b Spinal cord-DRG cultures were pretreated for 30 min with vehicle or 1 – 10 µM arimoclomol, followed by 30 min of heat shock at 43°C and recovery at 37°C for **a** 6 hr or **b** 24 hr. Cultures were fixed and immunolabeled for expression of stress-inducible Hsp70 (HSPA1A). Presence of absence of Hsp70 labeling was assessed in a minimum of 75 motor neurons in each of 3 cultures per condition. A dose-related increase in the percentage of motor neurons expressing Hsp70 was measured.

c Cultures were pretreated for 30 min with vehicle or 4 µM arimoclomol, followed by heat shock at 43° for 30 min and recovery at 37°C for 6-72 hr. Cultures were fixed and immunolabeled for expression of stress-inducible Hsp70. (HSPA1A). Presence of absence of Hsp70 labeling was assessed in a minimum of 75 motor neurons in each of 3 cultures per condition. The percentage of motor neurons expressing Hsp70 was increased at 6 h and 24 hr, but had returned to control levels by 48 hr.

Presented are means ± S.E.M, with statistical significance evaluated by one-way ANOVA and Bonferroni post-hoc analysis: *$p<0.05$, **$p<0.01$, ***$p<0.001$, ****$p<0.0001$.

Scale bar = 20 µm

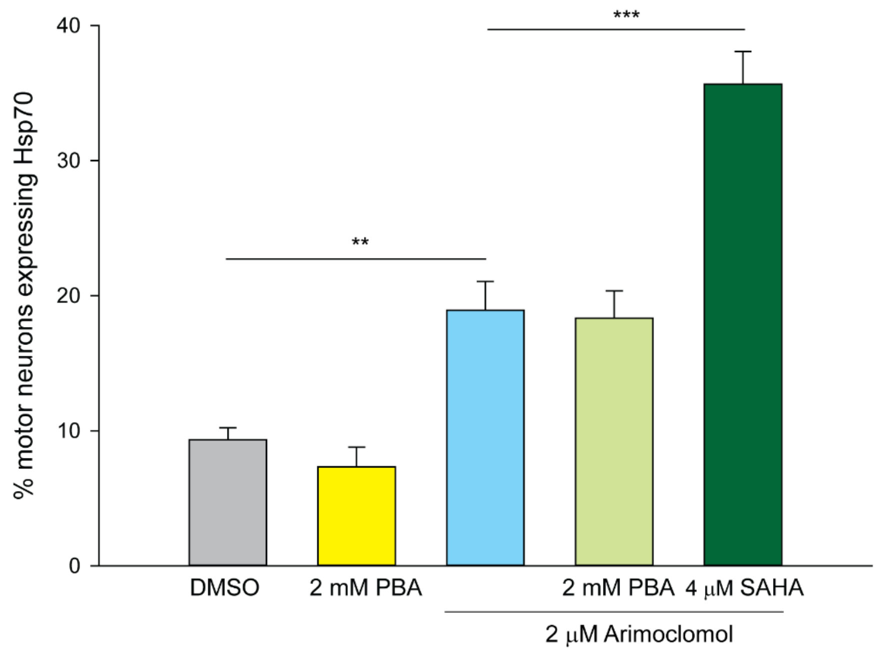

Online Resource 3 The pan HDAC inhibitor phenylbutyrate (PBA) failed to induce Hsp70 expression in heat shocked motor neurons or to potentiate arimoclomol or NXD30001. Murine spinal cord-DRG cultures were pre-treated for 30 min with vehicle (DMSO), 2 µM arimoclomol, 2 µM PBA or the combination of arimoclomol and phenylbutyrate, followed by heat shock at 43°C for 30 min and recovery at 37°C for 6 hr. Arimoclomol plus SAHA was included as a positive control for induction of Hsp70 (see Fig. 5a). Cultures were fixed and immunolabeled with antibody specific for stress-inducible Hsp70 (HSPA1A), followed by HRP-conjugated antibody and development using ImmPACT DAB kit (Vector Labs SK-4105. Presence of absence of Hsp70 labeling was scored in at least 75 motor neurons per culture. Presented are mean percentages ± S.E.M. of data collected from 3 cultures per condition. Statistical significance was evaluated by one-way ANOVA and Bonferroni post-hoc analysis: $*p<0.05$, $**p<0.01$, $***p<0.001$, $****p<0.0001$

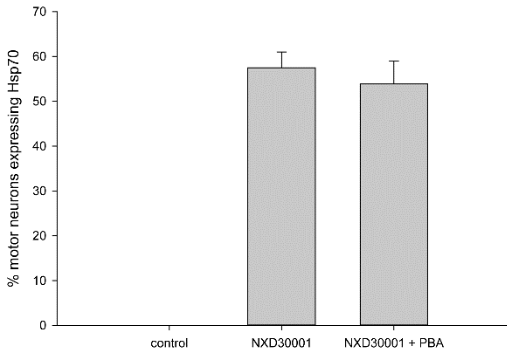

Phenylbutyrate (0.5 µM) failed to potentiate induction of Hsp70 expression in motor neurons by NXD30001 (40 nM)

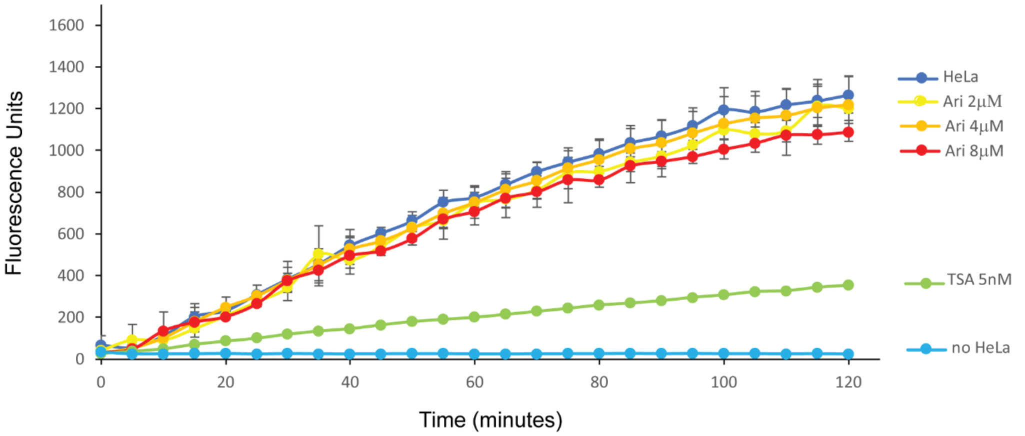

Online Resource 4

a Arimoclomol (Ari) did not affect activity of histone deacetylase (HDAC) class I enzymes *in vitro*. HDAC activity was assayed using the Abcam assay (Abcam ab156064) for HDAC class I activity (HDAC1, 2, 3 and 8). The assay was performed per manufacturer's instructions using Hela cell extracts supplied in the kit. The broad spectrum HDAC inhibitor, Trichostatin A (TSA), was used as a positive control at 5 nM. Assays were conducted in 96 well opaque plates (black wells) using 5 μl of crude nuclear extract from Hela, 5 μl of assay buffer, 5 μl of fluoro-acetylated peptide, 5 μl of developer (lysyl endpeptidase) (all provided in the kit) in a final volume of 50 μl). Arimoclomol was added to the wells at the indicated concentration. Fluorescence intensity was measured at intervals of 5 minutes for 2 hours using a microplate reader (SpectraMax M5, Molecular devices) at Ex/Em=355/460. Expressed are means of data from triplicate cultures ± S.D. Statistical analysis: 2-way ANOVA and Bonferroni post-hoc analysis. There was no significant difference ($p>0.05$) between samples containing arimoclomol and HeLa extract alone.

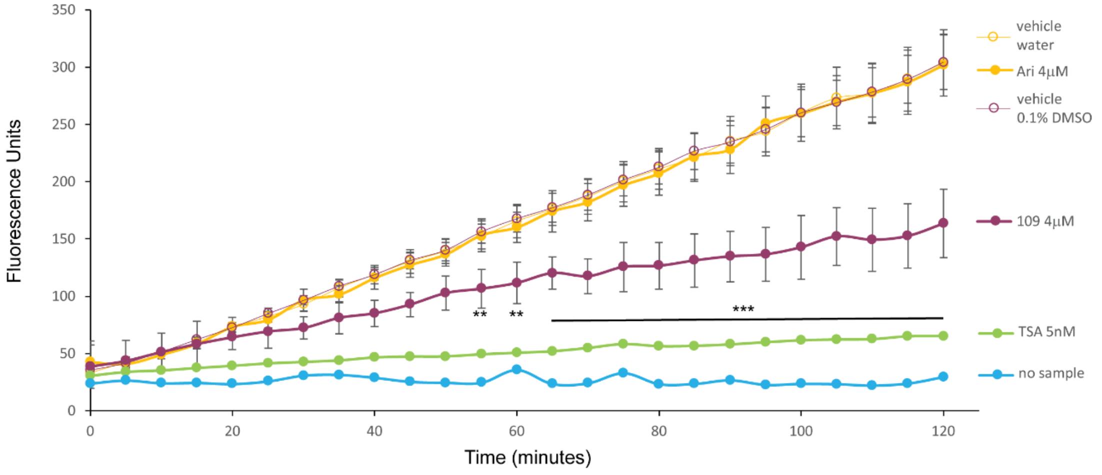

b Arimoclomol (Ari) and RGFP10 (109) were tested using total cell homogenates of spinal cord-DRG cultures, at the same concentration used in other experiments (4 μM). Arimoclomol had no significant effect on HDAC class I activity, whereas RGFP109 inhibited activity by 46%. Expressed are means of data from triplicate cultures ± S.D. Statistical analysis was carried out by 2-way ANOVA and Bonferroni post-hoc analysis. **$p<0.01$, ***$p<0.001$
